# Peptides from plants and microorganisms: a defense strategy for biotic and abiotic stresses

**DOI:** 10.3389/fpls.2025.1649512

**Published:** 2025-10-21

**Authors:** Dung Minh Ha-Tran, Chieh-Chen Huang

**Affiliations:** ^1^ Laboratory of Ecology and Environmental Management, Science and Technology Advanced Institute, Van Lang University, Ho Chi Minh City, Vietnam; ^2^ Faculty of Applied Technology, Van Lang School of Technology, Van Lang University, Ho Chi Minh City, Vietnam; ^3^ Department of Life Sciences, National Chung Hsing University, Taichung, Taiwan; ^4^ Advanced Plant and Food Crop Biotechnology Center, National Chung Hsing University, Taichung, Taiwan; ^5^ Innovation and Development Center of Sustainable Agriculture, National Chung Hsing University, Taichung, Taiwan

**Keywords:** antimicrobial peptides, biotic and abiotic stresses, salinity, drought, pathogenic fungi, plant diseases, insect pests, transgenic plants

## Abstract

Antimicrobial peptides (AMPs) from plants and microorganisms have emerged as promising tools due to their multifunctional roles in plant defense. These small, bioactive molecules, such as thionins, systemins, defensins, cyclotides, hevein-like peptides, and cyclic dipeptides, exhibit broad-spectrum activity against fungal pathogens, bacteria, and insect pests. Recent studies have further elucidated their supportive roles in conferring tolerance to abiotic stresses, including salinity, drought, and heavy metals exposure, thus expanding their potential applications. Previous studies demonstrated that the integration of AMPs genes into transgenic crops has shown significant potential in improving plant resistance to both biotic and abiotic stresses. Importantly, in our recent study, a cyclic dipeptide cyclo(L-Ala-Gly) from *Priestia megaterium* BP01R2 enables salinity stress alleviation in plants. The latest finding revealed that cyclo(His-Pro) in *Arabidopsis* navigated carbon flux from glycolysis to the pentose phosphate pathway and its supplementation increased NADPH levels and the NADPH/NADP^+^ ratio in plants. This review explores the latest advances in the application of plant- and microorganisms-derived AMPs, with a focus on their functional mechanisms and their roles in the development of stress-resilient crops. It also provides an overview of ongoing efforts to harness peptides in sustainable agricultural practices.

## 1 Introduction

The remarkable diversity, ubiquity, and versatility of antimicrobial peptides (AMPs) make them an abundant source of novel metabolites with potential applications in medicine, agriculture, and the food industry (Campos et al., 2018; Bakare et al., 2022; Simonsen et al., 2005). Various strategies have been implemented to alleviate the adverse effects of biotic and abiotic stresses on plants, including plant breeding (Hill et al., 1998), acclimation (Pandolfi et al., 2016), seed biopriming (Corbineau et al., 2023; Aizaz et al., 2023), plant growth-promoting rhizobacteria (PGPR) (Vaishnav et al., 2019), and the use of non-protein amino acids (NPAAs) (e.g., 5-hydroxynorvaline, meta-tyrosine, GABA, BABA, Canavanine, L-DOPA, Mimosine, etc.) (Silva Rodrigues-Corrêa and Fett-Neto, 2019). Research has also investigated the roles of various AMPs in plant defense systems to enhance resistance to phytopathogens (Maximiano et al., 2022) and to bolster adaptive mechanisms against abiotic stresses (Kulaeva et al., 2020). These studies show that AMPs can act directly as insecticidal molecules, inhibiting the growth of insect larvae (Mulla and Tamhane, 2023), or as inhibitors that suppress the growth of pathogenic bacteria or fungi (Huang et al., 2021).

Despite extensive investigation into their roles in suppressing bacteria and fungi (Leannec-Rialland et al., 2022; McLaughlin et al., 2021; Nawrot et al., 2014), the contributions of AMPs to mitigating the adverse effects of abiotic stresses remain less explored. Specifically, relatively few studies have examined the functions of AMPs in improving plant tolerance to salinity and drought stress (Li et al., 2021; Kumar et al., 2019; Hung et al., 2024), heavy metal toxicity (Mirakhorli et al., 2019), or wound stress (Rawat et al., 2017). This disparity in research emphasis can be attributed to several factors. Historically, AMPs were primarily identified and characterized for their antimicrobial properties, leading to a predominant focus on biotic stress responses, where their inhibitory effects on pathogens are straightforward to assay and quantify (Tang et al., 2023). In contrast, abiotic stresses, such as salinity and drought, involve complex physiological and molecular pathways (e.g., osmotic regulation or ion homeostasis), making it less intuitive and more challenging to evaluate the indirect effects or multifunctional roles of AMPs (Kulaeva et al., 2020). For instance, elucidating mechanisms of membrane permeability modulation and hormone signaling under abiotic conditions requires interdisciplinary approaches using transcriptomics, proteomics, and functional genomics, which demand specialized expertise and significant facilities (Deshmukh et al., 2025). Furthermore, abiotic stress experiments often necessitate controlled environments, which are more complicated than *in vitro* antimicrobial assays. While funding priorities have shifted toward abiotic stress research in recent years, driven by climate change concerns and the need for resilient crops, the focus remains on broader molecular mechanisms, leaving AMP-specific studies underexplored (Oyebamiji et al., 2024). This knowledge gap is critical, as emerging evidence suggests AMPs could enhance plant resilience through mechanisms like reactive oxygen species scavenging and hormone crosstalk, yet comprehensive studies remain sparse (Kaya and Adamakis 2025; González Ortega-Villaizán et al., 2024). Addressing this gap through targeted research could unlock novel applications for AMPs in sustainable agriculture. Our recent research, which demonstrated that the exogenous application of the cyclic dipeptide cyclo(L-Ala-Gly) contributes to salinity stress tolerance in plants, further highlights the potential of small bioactive molecules to enhance crop resilience against escalating global abiotic stresses (Hung et al., 2024). Beyond their direct application, AMP-encoding genes have also been widely incorporated into transgenic crops to confer new agronomic traits, including resistance to both biotic and abiotic stresses (Su et al., 2020; Parisi et al., 2020). To address this critical gap, this review synthesizes recent advances in the development of transgenic plants engineered with AMP-encoding genes from diverse plant and microbial sources. Furthermore, we explore the emerging potential of cyclic dipeptides to enhance the resistance of major crops to both biotic and abiotic stresses. By consolidating these findings, this manuscript offers valuable insights for researchers and agricultural scientists, guiding the development of novel, sustainable strategies to improve crop resilience and productivity in the face of diverse environmental challenges.

## 2 Classification and mechanisms of AMPs

### 2.1 Thionins

#### 2.1.1 Family and structure

**Figure 1:**
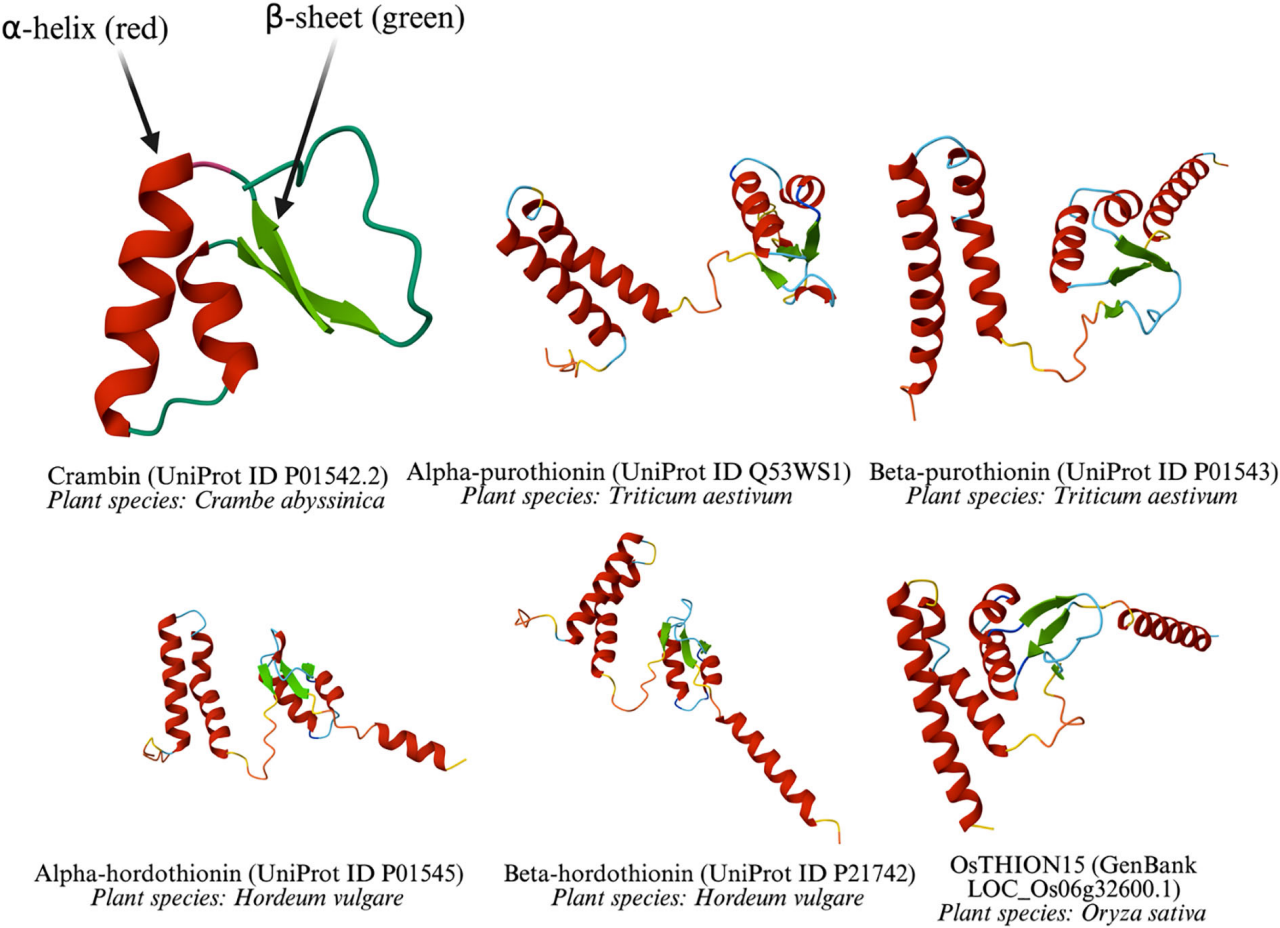
Three-dimensional structures of thionin from diverse plant species.

Thionins are a major class of plant-derived antimicrobial peptides (AMPs), alongside defensins, non-specific lipid transfer proteins, hevein-like peptides, knottin-like peptides, and cyclotides, distinguished by their cysteine spacing motifs and three-dimensional (3D) structures (Odintsova et al., 2018). As the first plant AMPs identified with *in vitro* antipathogenic activity, thionins are small peptides of approximately 5 kDa and 45–47 amino acids (aa), found predominantly in certain angiosperm families, including Poaceae (e.g., barley, wheat), Brassicaceae (e.g., *Arabidopsis*), and Ranunculaceae. Their compact structure, characterized by 6 or 8 cysteine residues forming 3 or 4 disulfide bridges, confers exceptional stability under diverse environmental conditions (Odintsova et al., 2018). Some common thionin structures were predicted using AlphaFold3 (Abramson et al., 2024) and are shown in **Figure 1**.

All depicted thionins share a common structural motif, including at least one α-helix and one β-sheet, which are critical for their antimicrobial function. These elements are stabilized by disulfide bonds, a hallmark of thionins, enabling them to disrupt microbial membranes. Differences in loop lengths and helix-sheet arrangements reflects species-specific variations, yet the conserved core structure underscores their shared evolutionary origin and functional role in plant defense. The diversity of plant species (*Crambe abyssinica*, *Triticum aestivum*, *Hordeum vulgare*, and *Oryza sativa*) highlights the widespread occurrence and adaptation of thionins across monocot and dicot plants.

#### 2.1.2 Mechanisms of antimicrobial action

Thionins primarily target pathogen cell membranes by interacting with negatively charged phospholipids, such as phosphatidic acid or phosphatidylserine, forming proteolipid complexes that disrupt membrane integrity through solubilization and lysis (Ji et al., 2015). This interaction also involves the extraction of phospholipids, reducing membrane fluidity and causing irreparable damage (Stec et al., 2004). Beyond their direct antimicrobial activity, thionins contribute to plant defense by activating signal transduction pathways mediated by phytohormones, including salicylic acid (SA), jasmonic acid (JA), and ethylene (ET) (Künstler et al., 2020). Previous studies further suggest that thionins enhance plant immunity by modulating reactive oxygen species (ROS) production and synergizing with other AMPs to bolster resistance against fungal and bacterial pathogens (Chan et al., 2005; Hao et al., 2020).

#### 2.1.3 Roles of thionins in biotic stress mitigation

##### 2.1.3.1 Antimicrobial activities

Thionins exhibit broad-spectrum activity against diverse biotic stressors, including bacteria, fungi, insects, and nematodes. A thionin-like peptide, CaThi, from *Capsicum annuum*, inhibited *Candida* spp. with half-maximal inhibitory concentrations (IC_50_) ranging from 10 to 40 µg/mL (Taveira et al., 2016). CaThi was shown to permeabilize the plasma membrane of all tested *Candida* spp. and induce oxidative stress in *Candida tropicalis*, with synergistic interactions with fluconazole significantly enhancing candidacidal activity (Taveira et al., 2016). Similarly, a thionin (*Thi2.1*) from *Arabidopsis thaliana*, when expressed in the BVE-E6E7 bovine endothelial cell line, reduced the viability of *Escherichia coli*, *Staphylococcus aureus*, and *Candida albicans* (Loeza-Ángeles et al., 2008). A cowpea thionin II expressed antifungal activity against *Fusarium culmorum* at minimum inhibitory concentration (MIC) ~ 50 µg mL^−1^, but *Aspergillus niger* and *Penecillium expansum* at MIC greater than 500 µg mL^−1^ (Schmidt et al., 2019). The thionin II activity remained at elevated temperature (100°C, 15 min) but lost its antifungal property in the presence of cations such as Na^+^, K^+^, Ca^2+^, and Mg^2+^ (Schmidt et al., 2019). A study by Moghadam et al. (2023) evaluated the antifungal efficacy of aqueous and alcoholic extracts from three varieties of *C. annuum* against various *Candida* species (Moghadam et al., 2023). The results, however, indicated that these extracts exhibited minimal inhibitory effects, with MIC exceeding 512 µg mL^−1^, suggesting limited antifungal activity in their crude form (Moghadam et al., 2023).

##### 2.1.3.2 Antifungal defense

A 2004 study by Oard et al. showed that, of 12 natural and synthetic AMPs, purothionin, a type I thionins from wheat seed, exhibited the strongest inhibitory activity against *Rhizoctonia solani* LR172, causal agent of rice sheath blight and aerial blight of soyabeans in the USA (Oard et al., 2004). Also in Oard et al. study, a strong correlation between membrane permeabilization and antifungal activity of the tested peptides was found with the most significant changes in membrane integrity at ≥ 0.5 µmol L^−1^ of purothionin, followed by 2 µmol L^−1^ of cecropin B, a natural peptide from cecropia moth (Oard et al., 2004). A 15 kDa thionin protein, Thi2.4, from *A. thaliana* was shown to interact with the fungal virulence factor fruit body lectin (FFBL), thereby reducing the toxicity of *Fusarium graminearum*. In addition to this protein–protein interaction, Thi2.4 exhibits direct antifungal activity by disrupting fungal cell membranes (Asano et al., 2013).

##### 2.1.3.3 Insect and nematode resistance

In barley (*Hordeum vulgare*), the thionin genes *THIO1567* and *THIO1570* exhibited significantly higher transcript levels in the bird cherry–oat aphid (*Rhopalosiphum padi* L.)-resistant genotype Hsp5 compared to the susceptible genotype Lina, suggesting a role in aphid resistance (Mehrabi et al., 2014). Similarly, in rice, thionin gene expression was more strongly suppressed in the susceptible genotype Nipponbare (*Oryza sativa*) than in the resistant accession TOG5681U (*Oryza glaberrima*) following nematode infection, indicating a potential contribution of thionins to nematode resistance (Petitot et al., 2017).

#### 2.1.4 Roles of thionins in abiotic stress mitigation

Under drought stress conditions, the susceptible barley cultivar Concerto showed increased expression of the *HvTHIO1* gene, suggesting a potential protective role (Leybourne et al., 2022). In rice, the defensin-dissimilar thionin gene *OsThi9* was strongly expressed in roots and stems under cadmium (Cd) exposure (0.1 µM CdCl_2_). Overexpression of *OsThi9* reduced Cd translocation to shoots, lowering Cd levels in leaves and grains (Liu et al., 2023). A combination of osmotic and heat stress caused higher susceptibility of *Arabidopsis* plants to *Botrytis cinerea*, possibly due to the reduced expression of defense genes, including *PLANT DEFENSIN 1.3 (PDF1.3)*, *BOTRYTIS SUSCEPTIBLE 1 (BOS1)*, *THIONIN2.2 (THI2.2)*, and cell wall-related genes (Sewelam et al., 2021). Recently, Yan et al. (2025) found that *OsTHION15* was significantly upregulated under drought stress, while the *Osthion15* mutant led to higher sensitivity to drought and abscisic acid (ABA) stress, indicating its key roles in abiotic stress resistance (Yan et al., 2025). While these studies indicate altered expression of thionin genes under abiotic stress conditions, such changes do not directly confirm the functional roles of thionins in stress tolerance. Therefore, further research is needed to elucidate the direct involvement of the thionins themselves in abiotic stress tolerance.

#### 2.1.5 Applications of thionin genes in transgenic plant development

*Thi2.1*, encoding 5 kDa cysteine-rich antimicrobial peptides, was upregulated in the resistant *Arabidopsis* ecotype UK-4 upon *Fusarium oxysporum* f. sp. *matthiolae* infection. Its overexpression in susceptible Col-2 seedlings delayed chlorophyll loss, inhibited fungal growth, and triggered severe fungal phenotype abnormalities (Epple et al., 1997). When *Thi2.1* was introduced into the tomato genome, the resulting transgenic plants showed enhanced resistance to Fusarium wilt (FW) (*F. oxysporum* f. sp. *lycopersici*) and bacterial wilt (BW) (*Ralstonia solanacearum*) (Chan et al., 2005). These *Thi2.1* transgenic lines matched the BW-resistant variety H7996 in disease incidence. Similarly, *Thi2.1* transgenic lines R7 and R11 showed a disease resistance comparable to the FW resistant variety MH1 and significantly lower disease incidence than WT plants (Chan et al., 2005). Meanwhile, β-purothionin, a 45-aa wheat endosperm thionin with 4 disulfide bonds and a strong cationic charge, offers environmental stability (Oard and Enright, 2006; Kaas et al., 2010; Stec, 2006). Transgenic *Arabidopsis* expressing *β-purothionin* gene driven by a leaf-specific chloroplast carbonic anhydrase promoter showed top-tier resistance to *Pseudomonas syringae* strain DC3000, with no leaf infection symptoms. *In vitro* tests against *F. oxysporum* revealed transgenic seedlings surviving 12–15 days with minimal necrosis and discoloration, while control died within 6–8 days (Oard and Enright, 2006). Thio-60 extracted from transgenic onion (*Allium cepa* L.) outperformed its non-transgenic counterpart, inhibiting *A. niger* spore germination by 52% versus 37% (Tawfik et al., 2022). A barley α-hordothionin (*α-HT*) gene with 384 bp in length, driven by the constitutive *El2Ω* or β-amylase (β*-Amy*) promoter (the 5’-UTR region), was transformed into sweet potato cultivar Kokei No. 14, to tackle *Ceratocystis fimbriata*, the top postharvest disease of sweet potato (Muramoto et al., 2012). *C. fimbriata* causes the most damaging postharvest disease of sweet potato (*Ipomoea batatas* (L.) Lam.) worldwide (Zhang et al., 2018). Wounds on storage roots were inoculated with a suspension of *C. fimbriata* spores to examine the resistance. Transgenic lines *El2Ω: α-HT* No. 1 and β*-Amy: α-HT* No. 060201 had much smaller black lesions than non-transgenic Kokei No. 14, with lesion areas of 119 mm^2^ and 111 mm^2^ compared to 283 mm^2^, respectively, indicating that the transgenic lines acquired solid resistance (Muramoto et al., 2012). In non-transgenic rice, *OsTHI7* normally exhibited root tip-specific expression, but its promoter activity was not detectable inside *Meloidogyne graminicola*-induced galls (Ji et al., 2015). This down-regulation might be a strategy by *M. graminicola* to suppress plant defense, thereby preventing the thionins from reaching toxic levels against the nematode. In contrast, transgenic rice lines that overexpressed *OsTHI7* showed decreased susceptibility to *M. graminicola* infection. These lines exhibited a significant reduction, approximately 39.2%, in the number of females and total nematodes per plant compared to control plants (Ji et al., 2015). This suggests a protective effect of OsTHI7 against the nematode, likely due to its direct toxic effect, as it possesses characteristics to alter cell membrane permeability and contribute to toxicity. Similarly, overexpression of *OsTHI7* in rice decreased susceptibility to *Pythium graminicola* colonization (Ji et al., 2015). Transgenic line OX22 displayed significantly healthier shoots with a lower disease rate and greater shoot length compared to control plants. Quantitative PCR analysis revealed that the quantity of *P. graminicola* DNA was significantly lower in the roots of *OsTHI7*-overexpressing lines (70% of total DNA) compared to wild-type and empty vector controls (97% and 98% respectively) (Ji et al., 2015).

Huanglongbing (HLB), the deadliest threat to global citrus production, has spurred extensive resistance engineering (Hao et al., 2017). A modified citrus thionin gene (*Mthionin*) gene, derived from an endogenous citrus thionin, was inserted into the Carrizo citrus genome. The transgenic Carrizo plants resisted HLB disease (*Candidatus Liberibacter asiaticus*) (Las) and citrus canker disease (*Xanthomonas citri*) (Hao et al., 2016). Leaf infiltration assays showed *Mthionin* transgenic leaves with little to no canker at *X. citri* concentration of 10^4–^10^7^ CFU mL^−1^. Grafted plants with transgenic Carrizo rootstock had significantly lower Las titers in young leaves and roots than controls, indicating that *Mthionin* is a promising HLB fighter (Hao et al., 2016). In another study, *Mthionin* gene, driven by a double *35S* promoter, was inserted into the *A. thaliana* genome (Hao et al., 2020). Lines A24 and A52, with peak *Mthionin* expression, showed reduced water soaking, lesions, and fungal biomass in detached leaf assays. Sprayed with *F. graminearum* (5 x 10^5^ conidia mL^−1^), *GUS* transgenic plants suffered severe symptoms, such as dry flowers, dry siliques, and dead branches, while *Mthionin* plants remained symptom-free. Furthermore, *F. graminearum* conidia barely germinated on *Mthionin* leaves. At 48 h post-inoculation, the *DEFENSIN1.2* expression in *Mthionin* plants was significantly higher than in *GUS* plants, hinting that *Mthionin* bolsters resistance to *F. graminearum* via defense genes and phytohormone signaling. Ectopic expression of two barley thionin genes *AK252675.1* and *AK359149* in *Nicotiana benthamiana* reduced host susceptibility to *Myzus persicae*, underscoring the important role of thionin genes against aphids (Escudero-Martinez et al., 2017). The *Thio-60* gene from *A. thaliana* was transformed into date palm (*Phoenix dactylifera* L.) cultivars Barhy, Sakkoti, and Shamia, yielding transgenic plants with higher resistance to *F. oxysporum*, confirmed by detached leaf pathogenicity test (Allah et al., 2023). In a more recent advancement, thionin genes (e.g., *thio-60* and *thio-63*) were introduced into Paulownia trees using chitosan nanoparticles as a delivery system for genetic transformation. The resulting transgenic lines exhibited enhanced resistance to fungal pathogens. Specifically, *thio-60* transgenic lines increased resistance to *Fusarium equiseti*, while *thio-63* transgenic lines enhanced resistance to *A. niger* (Bouqellah et al., 2024).

The integration of thionin genes into transgenic plant systems reveals consistent patterns of enhanced resistance across diverse biotic stressors and crop species. In response to fungal pathogens such as *Fusarium* spp., *A. niger*, *C. fimbriata*, and *F. graminearum*, overexpression of thionin variants (e.g., *Thi2.1*, *β-purothionin*, *α-HT*, *Mthionin*, *Thio-60*) has been shown to significantly reduce lesion sizes in sweet potato, inhibit spore germination in onion, delay seedling mortality *A. thaliana*, and produce symptom-free phenotypes. These effects are primarily attributed to direct antimicrobial activity and the upregulation of defense-related genes.

Comparable efficacy is observed under bacterial stress conditions, including infections by *R. solanacearum*, *P. syringae*, *X. citri*, and *Ca. L. asiaticus*. Transgenic lines exhibit reduced pathogen titers, absence of visible symptoms, and resistance levels on par with commercial cultivars. Although less frequently reported, insect resistance has also been demonstrated, notably against *M. persicae* in *N. benthamiana*, where ectopic thionin expression decreases host plant susceptibility. While model species such as *A. thaliana* and *N. benthamiana* continue to serve as valuable platforms for mechanistic studies, economically important crops—tomato, sweet potato, citrus, onion, date palm, and *Paulownia* spp.—exhibit promising translational outcomes, including postharvest protection and reduced disease incidence under laboratory- and field-relevant conditions. Collectively, these findings underscore the potential of *thionin* genes to confer broad-spectrum biotic resistance through membrane disruption and activation of defense pathways, supporting their utility in multi-pathogen engineering strategies across diverse plant systems.

### 2.2 Defensins

#### 2.2.1 Family and structure

**Figure 2:**
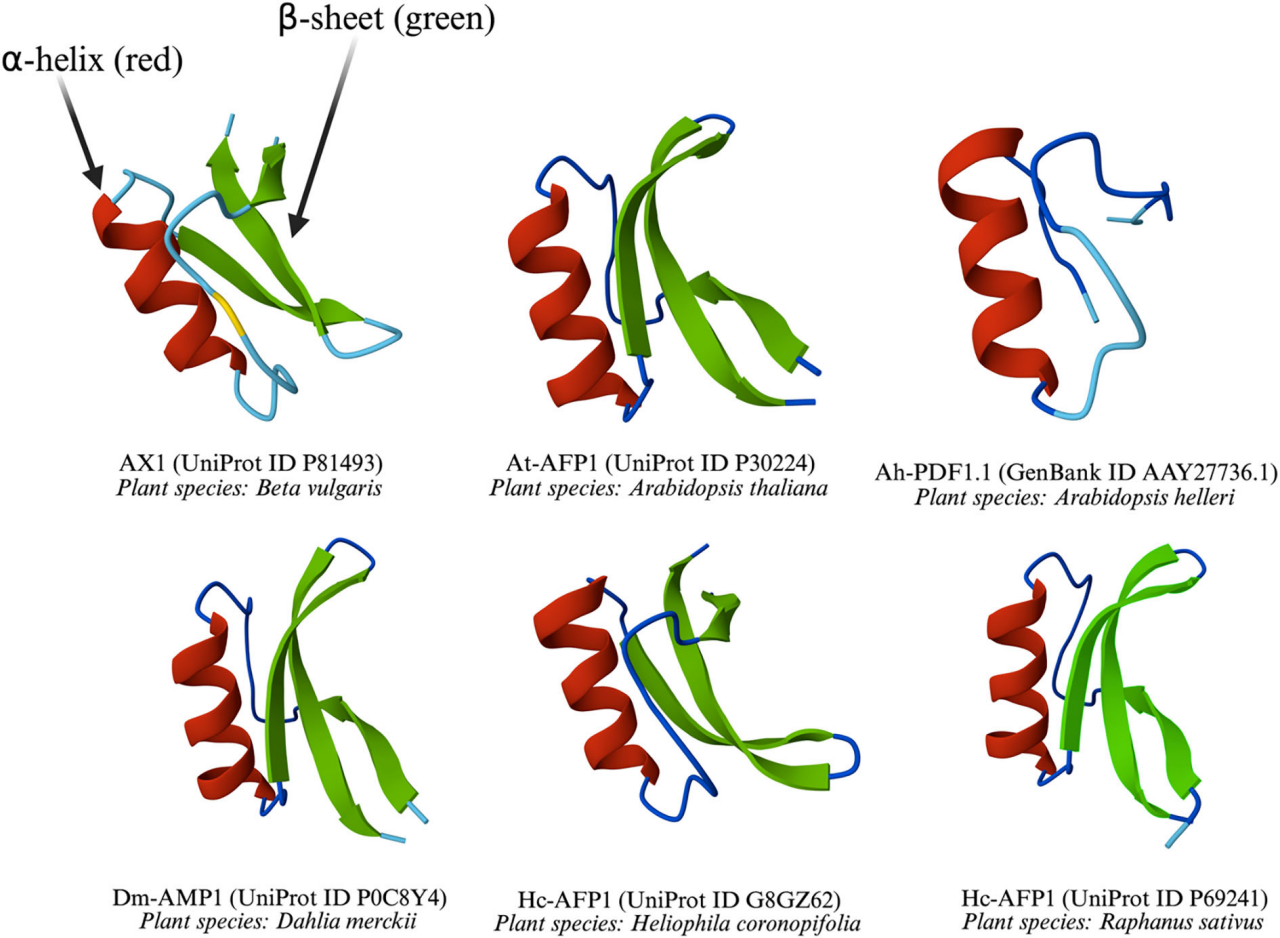
Three-dimensional structures of defensins from diverse plant species.

Like thionins, defensins are a key family of small, cysteine-rich, cationic peptides, but they are distinguished by a different structural architecture. They typically range from 45 to 54 aa, and feature eight conserved cysteine residues forming four disulfide bridges, which confer stability against protease degradation, extreme pH, and high temperatures (Azmi and Hussain, 2021; Kovaleva et al., 2020). The disulfide bonds cysteine residues in plant defensins possess the same pattern: Cys1-Cys8, Cys2-Cys5, Cys3-Cys6, and Cys4-Cys7 (Leannec-Rialland et al., 2022) with few exceptions (Janssen et al., 2003; Lay et al., 2003). Found across plant families such as Brassicaceae, Fabaceae, and Solanaceae families, plant defensins belong to the cis-defensin superfamily, distinct from the trans-defensin superfamily of mammals (Parisi et al., 2019). GMA4CG_V6, derived from MtDef4—a 47-aa defensin from *Medicago truncatula*—exhibited a multifaceted mode of action and antifungal activity against *B. cinerea*. Its 17-aa frame, containing the gamma-core motif of MtDef4, retains potent antifungal activity even at elevated salt concentrations (Tetorya et al., 2023). A characteristic γ-core motif (GXCX3–9C) is critical for their antimicrobial activity (Slezina et al., 2022; 2023; Sonderegger et al., 2018). The γ-core motif also plays role in mediating the entry of defensins into fungal cells (Sagaram et al., 2013; Tetorya et al., 2023). The Scots pine (*Pinus sylvestris* L.) defensin PsDef5.1 features a cysteine-rich α-motif (CX5CX3CX7CX9CXC) and a γ-core motif (GXCX9C), underscoring its structural complexity (Shalovylo et al., 2021). PsDef2 (RMCKTPSAKFKGYCVSSTNCKNVCRTEGFPTGSCDFHITSRKCYCYKPCP) and PsDef1 (RMCKTPSGKFKGYCVNNTNCKNVCRTEGFPTGSCDFHVAGRKCYCYKPCP), both defensins derived from *P. sylvestris*, differ by six aa in their primary sequences (Bukhteeva et al., 2022). Although this significant divergence is notable, with approximately 10–13% of the sequence, did not strongly affect their biological activities, including antimicrobial, antibacterial, and insect α-amylase inhibitory effects, implying the structural core, likely the cysteine-stabilized α-β (CSαβ) fold typical of plant defensins, is preserved (Bukhteeva et al., 2022). The CSαβ fold is likely critical for structural stability, functional scaffold, and dynamic flexibility of plant defensins. Some common defensin structures were predicted using AlphaFold3 (Abramson et al., 2024) and are shown in **Figure 2**.

All the defensins share a common structural motif, including at least one α-helix and one β-sheet, which are stabilized by disulfide bonds typical of defensins. These elements are critical for their ability to disrupt microbial membranes. The variation in loop lengths and helix-sheet arrangements reflects species-specific adaptations, while the conserved core structure underscores their shared evolutionary role in plant defense. The diversity of plant species such as *Beta vulgaris*, *A. thaliana*, *Arabidopsis helleri*, *Dahlia merckii*, *Heliophila coronopifolia*, and *Raphanus sativus* highlights the widespread occurrence of these defensins across different plant families.

#### 2.2.2 Mechanism of action

**Figure 3:**
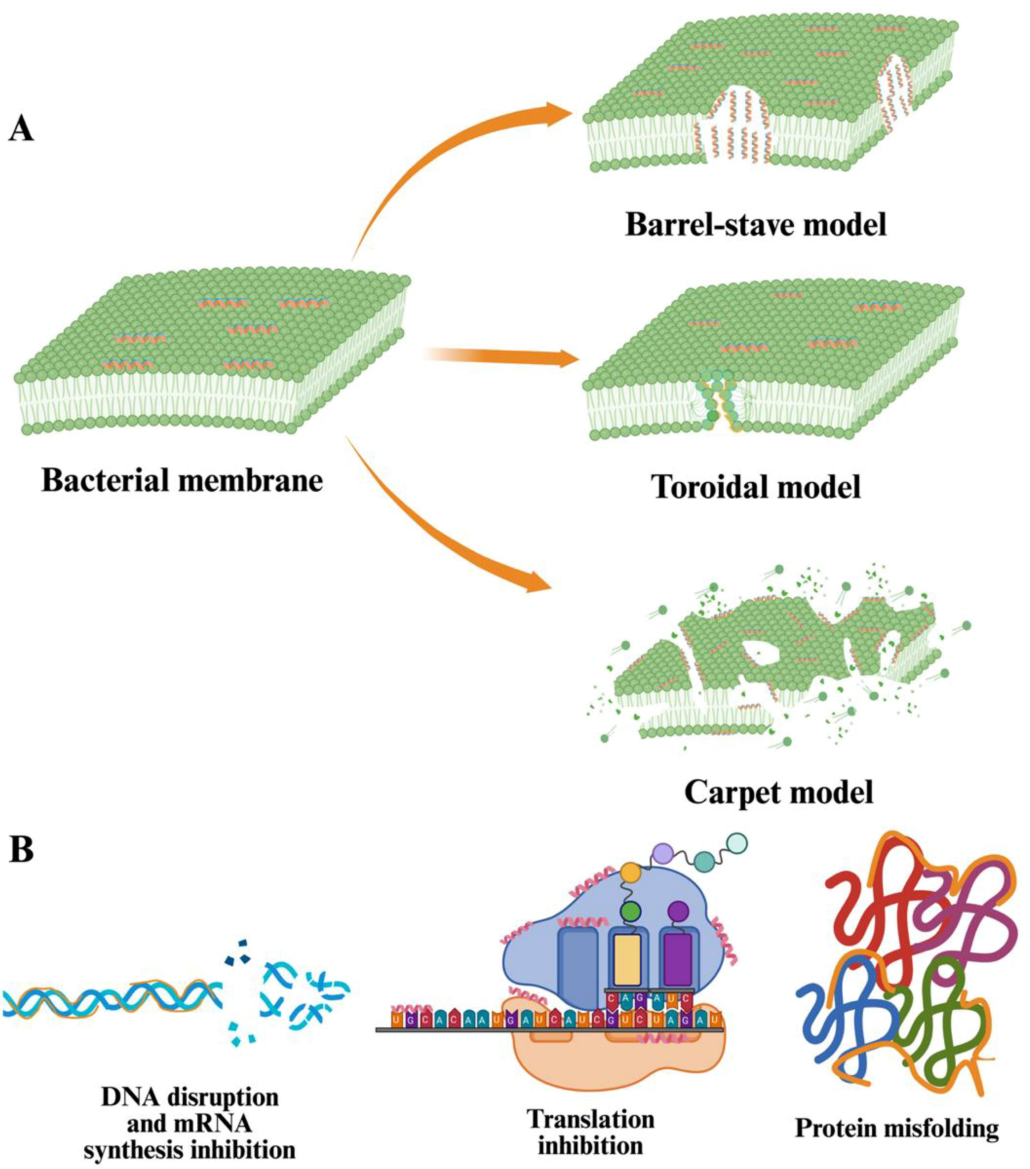
Two modes of action of AMPs. **(A)** In the barrel-stave model, the attached AMPs insert perpendicularly into the bacterial membrane. Their hydrophobic surfaces (shown in orange) bind to the lipid core of the bilayer, and their hydrophilic surfaces (shown in blue) form cylindrical pores, disrupting the membrane integrity. In the toroidal model, AMPs insert themselves into the lipid bilayer and induced the membrane to bend inward, creating a pore lined by both AMPs and the lipid headgroups. This results in a toroidal structure, where the lipids are significantly distorted. In the barrel-stave model, AMPs alone form a rigid pore structure, while the toroidal model triggers the interaction between the peptides and the lipids. In the carpet model, AMPs cover the bacterial membrane surface in a manner similar to a carpet. Initially, the AMPs do not form distinct pores. As the concentration of AMPs increases, they align parallel to the membrane surface and interact with the lipid headgroups through electrostatic and hydrophobic forces. This accumulation disrupts the membrane through a detergent-like mechanism involving partial micellization. Finally, the membrane undergoes disintegration and fragmentation, causing the release of cellular components and subsequent bacterial cell death. **(B)** AMPs can pass through cellular barriers to attach to nucleic acids (DNA, mRNA). AMPs exhibit high binding affinity to single- or double-stranded nucleic acids, allowing them to break down DNA molecules and inhibit mRNA synthesis. The attachment of AMPs on the double-helix DNA structure inhibits mRNA synthesis and disrupts DNA integrity. Also, AMPs can cause the blockage of protein translation by targeting ribosomes and protein misfolding by forming a complex with DnaK, a critical protein functioning in stabilizing *de novo* proteins. **Figure 3** is reproduced and adapted from earlier studies (Le et al. 2017; Matsuzaki 2019).

Similar to thionins, defensins and other AMPs deploy one or both primary antimicrobial strategies: membrane disruption and inhibition of cellular machinery (**Figure 3**). Their amphipathic nature enables them to target pathogen membranes, while specific residues enhance their efficacy. DefSm2-D (KLCEKPSKTWFGNCGNPRHCGDQCK-SWEGVHGACHVRNGKHMCFCYFNCPQAE), an antifungal defensin from wild thistle (*Silybum marianum*): truncated versions like SmAPγ27-44 (WEGAVHGACHVRNGKHMC), with its Arg38 residue in the γ-core domain, exhibited potent antagonism against *F. graminearum* (MIC_50_ of 20 μM), outperforms SmAPα1-21 (KLCEKPSKTWFGNCGNPRHCG) (MIC_50_ of 32 μM) and SmAPα10-21 (WFGNCGNPRHCG) (MIC_50_ of 70 μM) (Sagaram et al., 2013). The presence of three cationic lysine (Lys) residues, one anionic glutamic acid (Glu), and a tryptophan (Trp) in SmAPα1–21 likely enhances its membrane-binding capacity. Moreover, its selective action—targeting fungal cell walls over host cells—hinges on compositional differences between pathogen and plant membranes (Fernández et al., 2021).

[A] In the barrel-stave model, the attached AMPs insert perpendicularly into the bacterial membrane. Their hydrophobic surfaces (shown in orange) bind to the lipid core of the bilayer, and their hydrophilic surfaces (shown in blue) form cylindrical pores, disrupting the membrane integrity. In the toroidal model, AMPs insert themselves into the lipid bilayer and induced the membrane to bend inward, creating a pore lined by both AMPs and the lipid headgroups. This results in a toroidal structure, where the lipids are significantly distorted. In the barrel-stave model, AMPs alone form a rigid pore structure, while the toroidal model triggers the interaction between the peptides and the lipids. In the carpet model, AMPs cover the bacterial membrane surface in a manner similar to a carpet. Initially, the AMPs do not form distinct pores. As the concentration of AMPs increases, they align parallel to the membrane surface and interact with the lipid headgroups through electrostatic and hydrophobic forces. This accumulation disrupts the membrane through a detergent-like mechanism involving partial micellization. Finally, the membrane undergoes disintegration and fragmentation, causing the release of cellular components and subsequent bacterial cell death. [B] AMPs can pass through cellular barriers to attach to nucleic acids (DNA, mRNA). AMPs exhibit high binding affinity to single- or double-stranded nucleic acids, allowing them to break down DNA molecules and inhibit mRNA synthesis. The attachment of AMPs on the double-helix DNA structure inhibits mRNA synthesis and disrupts DNA integrity. Also, AMPs can cause the blockage of protein translation by targeting ribosomes and protein misfolding by forming a complex with DnaK, a critical protein functioning in stabilizing *de novo* proteins. **Figure 3** is reproduced and adapted from earlier studies (Le et al., 2017; Matsuzaki, 2019).

#### 2.2.3 Roles of defensins in biotic stress

Defensins play important roles in plant immunity, with expression often triggered by the mitogen-activated protein kinase (MAPK) cascade via effector-triggered immunity (ETI) and MAMP-triggered immunity (MTI) in response to fungal and bacterial assaults (Contreras et al., 2020). In pepper, the defensin gene *CADEF1* remained silent in healthy leaves but surged under infection by *Xanthomonas campestris* pv. *vesicatoria* (Mee Do et al., 2004). In rice, *OsDEF7* and *OsDEF8* ramped up expression following *Xanthomonas oryzae* pv. *oryzae* infection (Weerawanich et al., 2018). *Arabidopsis* defensin-like (DEFL) genes spiked in leaves challenged by *Alternaria brassicicola* or *P. syringae*, likely via JA signaling (Tesfaye et al., 2013). The transcription factor *WRKY75* further amplifies this response, upregulating JA pathway genes like *ORA59* and *PDF1.2* in overexpressing plants, while their expression drops in *wrky75* mutants (Chen et al., 2021). Defensins also exhibit direct antimicrobial activity, for example, cowpea Cp-thionin II inhibited *S. aureus* (MIC ~ 128 μg mL^−1^), *E. coli* (MIC ~ 64 μg mL^−1^), and *P. syringae* (MIC ~ 42 μg mL^−1^) (Franco et al., 2006), while Scots pine PsDef5.1, fused with thioredoxin, suppressed pathogens like *Fusarium* sp*orotrichiella* and *Bacillus pumilus* (Shalovylo et al., 2021). In resistant wild chickpea (ICC17160), six defensin genes (*CaDEF1.1B*, *CaDEF2.4*, *CaDEF2.5a*, *CaDEF3*, *CaDEF5*, and *CaDEFL2*) showed heightened expression against *F. oxysporum* and *Rhizoctonia bataticola* compared to the susceptible JG62 variety (Nitnavare et al., 2023). In a recent study by Kalunke et al. (2025), the bi-domain defensin MtDef5 from *Medicago truncatula* demonstrated antifungal properties at very low concentrations (Kalunke et al., 2025). It consists of two single-domains, MtDef5A and MtDef5B, linked by a short peptide APKKVEP. Between these two domains, MtDef5B showed greater antifungal potency compared with MtDef5A, exhibiting potent broad-spectrum inhibitory activity against *Botrytis cinerea* (*Bc*), *Fusarium graminearum* (*Fg*), *Fusarium virguliforme* (*Fv*), and the oomycete *Phytophthora capsici* (*Pc*), with MICs ranging from 0.75 µM to 1.5 µM for these pathogens. In contrast, a variant called GMA5AC_V2, derived from carboxy-terminal γ-core motif of MtDef5A (GMA5AC), exhibited two-fold higher activity (MIC ~ 1.5 µM) than GMA5AC (MIC ~ 3 µM) against *Bc*. It was at least four-fold more potent than GMA5AC against *Colletotrichum gloeosporioides* (*Cg*), Fg, and *Fv*. Notably, while GMA5AC had a MIC value of >12 µM against Cg, GMA5AC_V2 had a MIC value of 3 µM against this pathogen (Kalunke et al., 2025). The GMA5AC_V2 potent antifungal activity could be attributed to the replacement of two cysteine residues (C12 and C14) with phenylalanine in its structure (Kalunke et al., 2025). Furthermore, GMA5AC_V2 also displayed a multi-faceted mode of action similar to MtDef5B as it disrupted the fungal plasma membrane, induced the production of ROS in both *Bc* and *Cg* germlings, and inhibited protein translation *in vitro* (Kalunke et al., 2025). In summary, plant defensins contribute to biotic stress mitigation by acting as both immune response regulators and direct antimicrobial agents, enhancing resistance against a broad spectrum of fungal and bacterial pathogens.

#### 2.2.4 Roles of defensins in abiotic stresses

##### 2.2.4.1 Defensin-mediated mechanisms for heavy metal tolerance

A primary mechanism for heavy metal tolerance is the chelation of metal ions by the cysteine-rich domains of defensins, followed by the efflux of the metal-defensin complex from the cytoplasm to less sensitive extracellular compartments like cell walls or the apoplast, or into vascular tissues for transport (Luo et al., 2018; Luo et al., 2019; Li et al., 2025; Luo et al., 2019). Defensins mitigate abiotic stresses through these mechanisms, which include metal chelation, regulation of metal transport, and modulation of physiological responses. Many defensins (e.g., CAL1, DEF8, AtPDF1.5, AtPDF2.5, NtCAL1) are secreted to the cell wall or extracellular spaces (Luo et al., 2018; Gu et al., 2023; Liu et al., 2021; Luo et al., 2020, 2019; Gu et al., 2023; Jin et al., 2024), while others, like AtPDF2.6 and SpPDF, are localized in the cytoplasm, where they directly chelate metals to mitigate toxicity (Luo et al., 2019; Li et al., 2025).

##### 2.2.4.2 Specific roles of defensins in Cd stress mitigation

Cd is a major heavy metal pollutant posing significant threats to food security and human health, making Cd detoxification and accumulation a critical area of study (Luo et al., 2018; Luo et al., 2019; Li et al., 2024; Luo et al., 2020). Several defensins play key roles in managing Cd stress by regulating its chelation, transport, and accumulation in different plant tissues. Some defensins are regulators of root-to-shoot translocation, acting as secreted proteins that primarily control the movement of Cd from roots to shoots. For example, CAL1 (Cd Accumulation in Leaf 1) in rice is a cell wall-localized defensin-like protein (Luo et al., 2018) that positively regulates Cd accumulation in leaves (Luo et al., 2018; 2020). It chelated Cd in the cytosol and drove its secretion to extracellular spaces, promoting its loading into xylem vessels for root-to-shoot translocation and accumulation in leaves and straws, but not in rice grains (Jin et al., 2024). Similarly, *NtCAL1* in tobacco functions as a positive factor in plant Cd accumulation and resistance (Jin et al., 2024), with its overexpression enhancing Cd translocation to the shoot. Overexpression of *NtCAL1* enhanced the content of ascorbic acid and the activities of antioxidant enzymes such as catalase (CAT) and ascorbate peroxidase (APX), helping to scavenge oxidative stress induced by Cd (Jin et al., 2024). It also positively regulated the expression of metal transport-related genes such as *NtRAMP3, NtHMAα, NtHMAβ* (Jin et al., 2024). *AtPDF2.5* in *Arabidopsis* mediated Cd tolerance and accumulation (Luo et al., 2019), and its expression, which is induced by Cd, was primarily expressed in root xylem vascular bundles (Luo et al., 2020). This defensin has Cd-chelating activity (Luo et al., 2019) and promotes cytoplasmic Cd efflux and subsequent accumulation in the apoplast of cell walls, thereby enhancing detoxification and apoplastic accumulation (Luo et al., 2020). Additionally, *OsThi9*, a defensin-dissimilar thionin, alleviates Cd toxicity by sequestering Cd in cell walls and reducing its translocation to upper parts, which diminishes Cd accumulation in stems and brown rice (Liu et al., 2023). Other defensins act as modulators of shoot and grain accumulation, controlling Cd allocation within the plant. *CAL2*, a close homolog of *CAL1*, is a cell wall-localized protein with Cd chelation activity. Overexpression of *CAL2* has been shown to increase Cd accumulation in both rice shoots and grains (Luo et al., 2020). Its expression is unaffected by Cd stress but is positively regulated by the endoplasmic reticulum stress response regulator *OsbZIP39* (Li et al., 2024). DEFENSIN 8 (DEF8) in rice is a dual-function protein that mediates both xylem Cd loading and phloem Cd unloading (Gu et al., 2023). It was highly expressed in rice grains and was induced by Cd exposure (Gu et al., 2023). While DEF8 facilitates Cd transport from roots to shoots, it prevents Cd loading from the phloem into the grains during filling, making it an ideal target for breeding low-Cd rice varieties (Kazachkova 2023). *AtPDF1.5* enhanced adaptation to low nitrogen levels and Cd stress in *Arabidopsis* (Wu et al., 2021). Its mechanism for Cd tolerance may be attributed to chelation and signal transmission, as it regulates the expression of metal transporter genes such as *AtHMP07, AtNRAMP4, AtNRAMP1, AtHIPP3*, resulting in higher Cd accumulation in shoots and promoting Cd transport (Wu et al., 2021). Some defensins are cytoplasmic chelators that act within the cytoplasm to directly sequester metals. AtPDF2.6 in *A. thaliana* is a non-secreted, cytoplasm-localized protein that actively chelates cytoplasmic Cd to mitigate its toxicity (Luo et al., 2019). Similarly, *SpPDF* in *Sedum plumbizincicola*, a hyperaccumulator plant, is another cytoplasm-localized defensin that confers Cd accumulation via its chelation (Li et al., 2025). Its chelation activity leads to increased Cd accumulation in the roots and reduced translocation to the shoots, helping the plant compartmentalize metals away from sensitive aerial parts (Li et al., 2025).

##### 2.2.4.3 Roles of defensins in other abiotic stress mitigation

Apart from heavy metal responses, defensins actively contribute to abiotic stress responses, including drought, salinity, and heavy metal tolerance. The pepper *CADEF1* gene, for example, was activated by abiotic elicitors (e.g., H_2_O_2_, wounding, salinity, drought) and stress hormones like SA, Methyl Jasmonate (MeJA), JA, abscisic acid (ABA), and ET (Contreras et al., 2020). Similarly, rice *OsDEF7* and *OsDEF8* responded to imbibition, anoxia, drought, and cold (Weerawanich et al., 2018). In *Arabidopsis halleri* ssp. *halleri*, a zinc (Zn)-tolerant species, defensin proteins accumulated in shoots and gene expression increased under Zn exposure (Mirouze et al., 2006). *Arabidopsis AtPDF1.1* is upregulated by iron overload and *Pectobacterium carotovorum* infection, enhancing tolerance by chelating iron and disrupting pathogen homeostasis (Hsiao et al., 2017). These previous studies, however, did not directly confirm the functional roles of defensin genes in plant stress tolerance under various abiotic stresses, underscoring the need for further mechanistic studies to validate their contributions.

#### 2.2.5 Applications of defensin genes in transgenic plant development

Defensin genes have been harnessed to engineer stress-resistant crops, demonstrating their versatile applications against both biotic and abiotic challenges. The first attempt to transform defensin genes into plant genomes to increase resistance towards phytopathogenic fungi was made by Bondt et al. (1998) (Bondt et al., 1998). Subsequently, the expression of alfalfa antifungal peptide (*alfAFP*) gene, a defensin isolated from *Medicago* sativa seeds, in transgenic potato plants, rendered high levels of field resistance against *Verticillium dahliae*, the causative agent of “early dying” disease of potato (Gao et al., 2000). *In vitro* assays showed that alfAFP inhibited hyphal elongation of *V. dahliae*, *Alternaria solani* and *Fusarium culmorum*, but not *Phytophthora infestans*. The secretion of alfAFP in the intercellular spaces of stem tissues protected transgenic potato in both greenhouse and multi-year trials (Gao et al., 2000). Transgenic wheat (Bobwhite and Xin Chun 9 genotypes) expressing an apoplast-targeted antifungal plant defensin, MtDef4.2, from *Medicago truncatula* displayed significant resistance to the leaf rust pathogen *Puccinia triticina* (*Pt*) (Kaur et al., 2017). This resistance was observed in growth chamber bioassays, where transgenic lines showed highly resistant infection types compared to non-transgenic controls. Interestingly, while MtDef4.2 reduced the proliferation of infection hyphae and minimized lesion sizes on infected wheat leaves, it did not negatively affect symbiotic relationship between the host plant and the beneficial arbuscular mycorrhizal fungus *Rhizophagus irregularis* (Kaur et al., 2017). This is a significant advantage for its agricultural application.

While early efforts focused on introducing defensins to combat phytopathogenic fungi (Bondt et al., 1998; Wang et al., 1999; Lebedev et al., 2002; Cary et al., 2000; Park et al., 2002; Swathi Anuradha et al., 2008; Abdallah et al., 2010), recent applications have expanded to include environmental stressors. Overexpression of the *NaD1* gene from *Nicotiana alata* in tobacco (*Nicotiana tabacum*) enhanced drought tolerance by maintaining photosynthetic pigments and boosting antioxidant enzyme activity, which reduces oxidative damage (Royan et al., 2023). In the context of heavy metal tolerance, the *AhPDF1.1* defensin from a Zn-tolerant species, when transformed into *Arabidopsis*, conferred increased Zn tolerance (Mirouze et al., 2006). Further findings highlight the multifaceted roles of defensins in heavy metal mitigation. Overexpression of *AtPDF2.5* significantly enhanced tolerance and accumulation of Cd in both the host *Arabidopsis* and in rice through heterologous expression. This suggests that *AtPDF2.5* promotes cytoplasmic Cd efflux via chelation, thereby enhancing detoxification and apoplastic accumulation (Luo et al., 2019). Similarly, *AtPDF2.6* overexpression also enhanced Cd tolerance, while its knockout increased Cd sensitivity, emphasizing its role in detoxification via chelation and efflux (Luo et al., 2019). Beyond direct chelation, defensins can be regulated by stress-signaling pathways. The *OsbZIP39* transcription factor, a key regulator of the unfolded protein response in the endoplasmic reticulum, positively regulates Cd accumulation in rice. Upon Cd exposure, *OsbZIP39* is activated and enhances the expression of the defensin-like protein OsCAL2, which in turn facilitates Cd accumulation in the plant (Li et al., 2024). These findings show that defensin-based strategies can be leveraged to develop crops with resilience to various environmental stresses through distinct mechanisms, including antioxidant activity, metal chelation, and ion efflux.

### 2.3 Cyclotides

#### 2.3.1 Family and structure

Cyclotides represent another fascinating class of AMPs with a unique, highly stable structure, which enables them to resist harsh environmental conditions. This is a salient group of plant-derived macrocyclic peptides, typically containing 28–37 aa and featuring an embedded cystine knot. These peptides have been identified in five major plant families, including Rubiaceae, Violaceae, Solanaceae, Cucurbitaceae, and Fabaceae (Slazak et al., 2020). Cyclotide-like genes have also been identified in members of the Poaceae family, including *O. sativa*, *Zea mays*, *T. aestivum*, *Agrostis stolonifera*, *Schedonorus arundinaceus*, *Pennisetum glaucum*, *Sorghum bicolor*, *Hordeum vulgare*, *Saccharum officinarum*, and *Setaria italica*. Cyclotides are distinguished by their unique structural characteristics, which include a head-to-tail cyclic peptide backbone and a cystine knot motif, where two disulfide bonds are interlinked by a third. This structural conformation confers exceptional stability, enabling cyclotides to remain functional under extreme temperatures, pH variations, and enzymatic degradation while maintaining solubility in both organic and aqueous environments (Veer et al., 2019).

**Figure 4:**
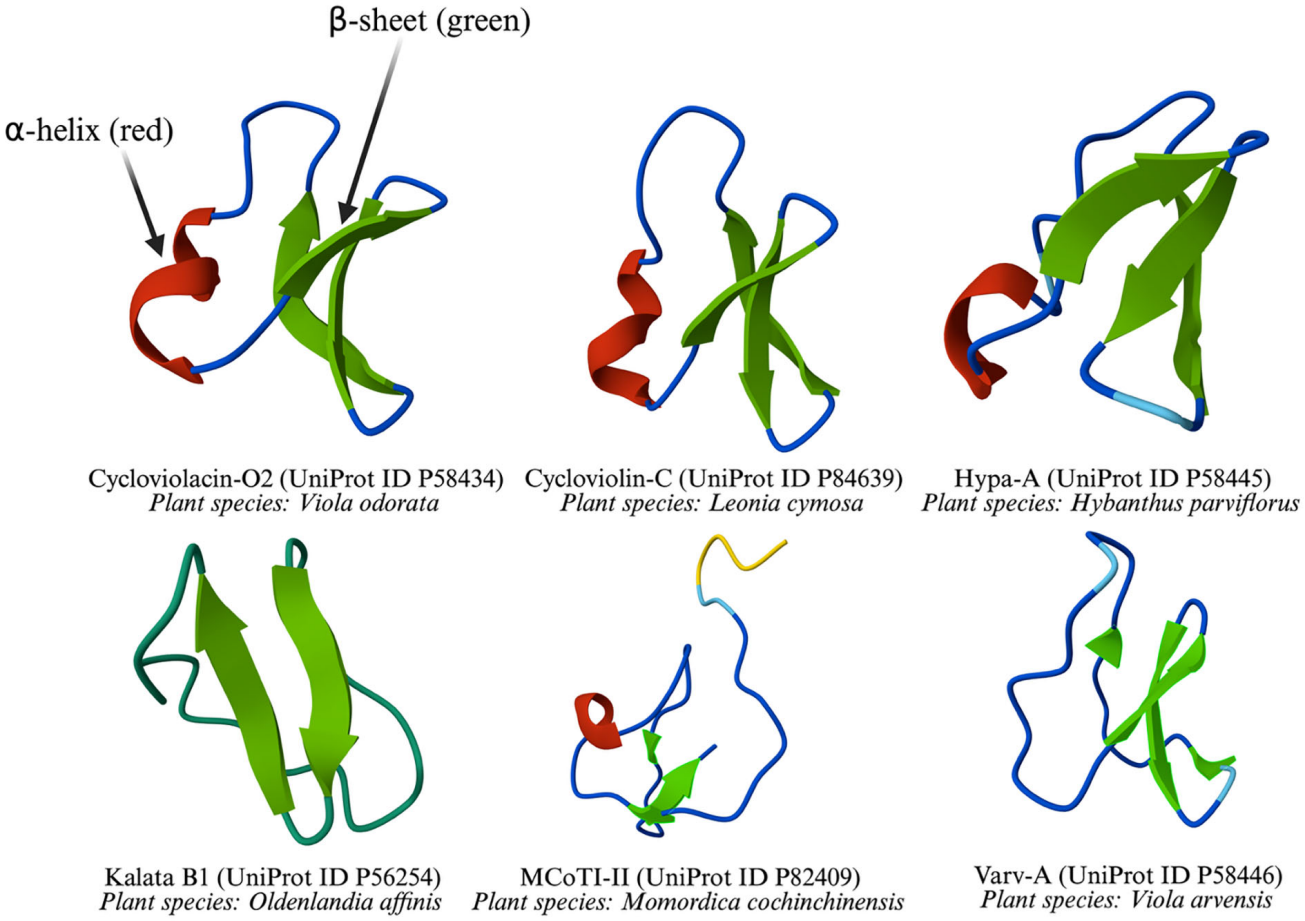
Three-dimensional structures of cyclotides from various plant species.

One of the most well-characterized cyclotides, kalata B1, was initially isolated from *Oldenlandia affinis* and has since been identified in several *Viola* species, including *Viola tricolor*, *Viola yedoensis*, *Viola philippica*, *Viola baoshanensis*, and *Viola odorata*. It was the first cyclotide to have its structure elucidated (Saether et al., 1995; Veer et al., 2019). The exceptional stability of cyclotides is attributed to their cyclic peptide backbone and cystine knot motif, which enable them to withstand extreme conditions such as high temperatures and enzymatic degradation within the human digestive system. The stability of kalata B1 has been linked to these structural features, which historically contributed to the use of *O. affinis* as a medicinal tea in Congolese tribes, where it was known as “kalata-kalata” in the Tsjiluba language and used to facilitate childbirth (Gran et al., 2000). Some cyclotides structures were predicted using AlphaFold3 (Abramson et al., 2024) and shown in **Figure 4**.

The figure illustrates the 3D-structures of six common cyclotides, which are characterized by one or more β-sheets, with and without α-helix. These representations highlight structural diversity across different plant species.

#### 2.3.2 Mechanism of action

As with other AMP classes, cyclotides exert their bioactivity through multiple mechanisms, including membrane disruption and targeting of insect physiology. Cycloviolacin-O2 (CyO2) from *V. odorata* exhibits antimicrobial properties by disrupting cellular membranes and interfering with intracellular targets, with MICs ranging from 0.8 to 100 µM against fungal and bacterial pathogens such as *F. oxysporum*, *Botrytis cinerea*, and *P. syringae* (Oguis et al., 2019). Additionally, insecticidal cyclotides such as kalata B1 and kalata B2 target the physiological integrity of insect midgut cells. Kalata B1, for example, induces rupture of epithelial cells in the midgut of *Helicoverpa armigera* larvae, a mode of action similar to that of *Bacillus thuringiensis* δ-endotoxins (English and Slatin, 1992; Barbeta et al., 2008). Furthermore, the high metal-binding affinity of cyclotides suggests their potential involvement in detoxification processes, though the underlying mechanisms require further investigation (Zhang et al., 2015). The paper by Miszczak et al. (2025) tested whether differences in cyclotide production between metallicolous (M) and non-metallicolous (NM) populations under Zn and lead (Pb) stress and their functions in alleviating metal stress (Miszczak et al., 2025). In M population, the decrease in cyclotide production under heavy metal stress may be explained the reallocation of resources towards other tolerance mechanisms (e.g., metal sequestration or callose production), while in NM population, the increase in cyclotide production suggests an inducible defense response to mitigate oxidative stress or cellular damage caused by heavy metals. In these conditions, cyclotides may act as metal chelators or membrane stabilizers to protect metal-sensible plants (Miszczak et al., 2025). However, the paper did not provide direct evidence of cyclotide function, such as metal binding, detoxification, or cellular protection, leaving their mechanistic role unclear.

#### 2.3.3 Roles of cyclotides in biotic stress

Cyclotides, like thionins and defensins, serve as potent natural defenses against biotic stressors. Kalata B1 and B2, derived from *O. affinis*, exhibited significant insecticidal activity. Kalata B1 effectively inhibited the growth of *Helicoverpa punctigera* and *H. armigera* larvae at a concentration of 0.13% (w/v), while reducing nutrient uptake at 0.24% (w/v) (Jennings et al., 2001; 2005). Kalata B2 demonstrated superior molluscicidal efficacy compared to metaldehyde in controlling the golden apple snail (*Pomacea canaliculata*), a major pest in rice cultivation, with an LC_50_ of 53 μM, whereas metaldehyde exhibited an LC_50_ of 133 μM (Plan et al., 2008). Unlike metaldehyde, which poses toxicity risks to non-target mammals, kalata B2 presents an environmentally safer alternative (Tan et al., 2022). Furthermore, the cyclotide Cter M, isolated from *Clitoria ternatea*, demonstrated efficacy against *H. armigera*, contributing to the development of Sero-X^®^, a bioinsecticide approved for commercial use in Australia in 2017 (Oguis et al., 2019). In terms of antimicrobial properties, cycloviolacin-O2 (CyO2) from *V. odorata* exhibits antifungal and antibacterial activity against pathogens such as *Colletotrichum utrechtense*, *Alternaria alternata*, and *Dickeya dadantii* (Oguis et al., 2019). Additionally, cyclotide-like genes in *Z. mays* (*Zmcyc1* and *Zmcyc5*) were reported to contribute to plant defense against fungal pathogens such as *F. graminearum* and insect pests like *Rhopalosiphum maydis*, suggesting a broader protective function in plant immunity (Salehi et al., 2017).

#### 2.3.4 Roles of cyclotides in abiotic stress

Emerging evidence suggests that cyclotides also contribute to abiotic stress tolerance, although their exact mechanisms remain underexplored. *V. baoshanensis*, a known Cd hyperaccumulator, accumulates Cd concentrations of 1168 mg kg^−1^ in shoots and 981 mg/kg in roots when grown in Pb-Zn mining areas (Liu et al., 2004). In response to Cd exposure, cyclotide precursor mRNAs *VbCP7S* and its spliced variant *VbCP6S* were upregulated in the leaves, indicating a potential role in metal binding and detoxification (Zhang et al., 2009). In *Z. mays*, cyclotide-like genes *Zmcyc1* and *Zmcyc5* are induced under abiotic stress conditions, including drought, salinity, and mechanical wounding. Their expression is also modulated by phytohormones such as SA and MeJA, suggesting involvement in stress response pathways linked to plant defense (Salehi et al., 2017). Additionally, studies have highlighted the strong metal-binding affinity of cyclotides, raising the possibility that they contribute to heavy metal detoxification, though further research is required to elucidate their precise function in abiotic stress adaptation (Zhang et al., 2015).

#### 2.3.5 Applications of cyclotide genes in transgenic plant development

Cyclotides present promising applications in transgenic plant development, demonstrating a dual role in conferring resistance to both biotic and abiotic stresses. In the context of abiotic stress, cyclotides show potential for heavy metal detoxification. For example, five cyclotide precursor genes (VbCP1–VbCP5) from *V. baoshanensis* enhanced copper (Cu) tolerance when expressed in *Saccharomyces cerevisiae* (Zhang et al., 2009), demonstrating their metal-binding capabilities. This approach is similar to the use of a chicken-derived metallothionein gene, which significantly improved Cd sequestration in *Chlamydomonas reinhardtii* (Cai et al., 1999). This highlights that cyclotides, like other metal-binding proteins, can be leveraged to increase tolerance to heavy metal contamination. In parallel, cyclotides have been successfully engineered for biotic stress resistance. The *kalata B1* (*Oak1*) gene from *O. affinis* was introduced into rice, resulting in transgenic plants that provided protection against a specific agricultural pest, the golden apple snail (Lim and Lai, 2017). These findings collectively underscore the exceptional versatility of cyclotides as valuable molecular tools for developing crops with improved resilience to both herbivorous pests and heavy metal contamination, using distinct mechanisms for each stress type. More recently, a study by Qu et al. (2020) successfully produced a structurally validated cyclotide in rice suspension cells in the presence of a supporting biosynthetic enzyme asparaginyl endopeptidase (AEP), which is derived from the *O. affinis* plant (Qu et al., 2020). This enzyme is crucial in performing the final, essential step in cyclotide biosynthesis—the head-to-tail cyclization of the linear precursor peptide (Tang and Luk 2021). Without it, the plant would only produce a linear peptide, not the highly stable, circular cyclotide. The study’s main finding was that co-expressing this enzyme with the cyclotide precursor gene was necessary to achieve the correct, functional structure of the kalata B1 cyclotide in the rice suspension cell (Qu et al., 2020). This research confirms that the biological machinery of a plant can be harnessed to correctly produce complex cyclotides, validating their potential for crop engineering.

### 2.4 Hevein-like antimicrobial peptides

#### 2.4.1 Family and structure

Hevein-like AMPs are a family of structurally related peptides characterized by their similarity to hevein, a peptide originally identified in the latex of the rubber tree (*Hevea brasiliensis*) (Van Parijs et al., 1991). These peptides are cysteine- and glycine-rich, typically containing 6–8 cysteine residues that form disulfide bonds, contributing to their stable, compact structures. They are found across various plant families, including both monocots and dicots, indicating their widespread distribution in the plant kingdom (Slavokhotova et al., 2017). A key structural feature of hevein-like AMPs is the chitin-binding domain, which facilitates interaction with chitin and related oligosaccharides, a critical component of fungal cell walls and insect exoskeletons. For instance, the 40-amino-acid Pn-AMP2 and the 8C-hevein-like ginkgotide gB5 from *Ginkgo biloba* exemplify this structural motif, which underpins their biological activities (Koo et al., 1998; Wong et al., 2016).

#### 2.4.2 Mechanisms of antimicrobial action

Hevein-like AMPs exhibit broad-spectrum antimicrobial activity through mechanisms primarily based on their chitin-binding capability. The chitin-binding domain allows these peptides to target chitin-containing structures in fungal cell walls, disrupting membrane integrity and inhibiting fungal growth. Pn-AMP2 demonstrated antifungal activity against both chitin-containing and non-chitin-containing fungi, suggesting additional mechanisms such as membrane permeabilization or interference with cellular processes (Koo et al., 1998). Similarly, the PMAPI protein from paper mulberry (*Broussonetia papyrifera*) inhibited *Trichoderma viride* with an IC50 of 0.1 μg/μL (Zhao et al., 2011). Additionally, some hevein-like AMPs, such as WAMP-1a from *Triticum kiharae*, exhibited antibacterial activity against Gram-positive and Gram-negative bacteria by disrupting bacterial cell membranes (Odintsova et al., 2009). These peptides also counteract fungal proteases, such as fungalysin from *Fusarium verticillioides*, which degrades plant chitinases, thereby preserving plant defense mechanisms (Slavokhotova et al., 2014).

#### 2.4.3 Roles in biotic stress mitigation

Hevein-like AMPs play a critical role in plant defense against biotic stresses, including fungal, bacterial, and insect challenges. Their antifungal properties are well-documented, with peptides like WAMP1 and WAMP2 from *Triticum kiharae* showing upregulated expression in response to *Fusarium oxysporum* infection and inhibiting fungal growth (Andreev et al., 2012). Ginkgotide gB5 from *Ginkgo biloba* inhibited fungi such as *A. niger* (IC_50_ ~ 6.8 μg mL^−1^) and *R. solani* (IC_50_ ~ 20 μg mL^−1^) (Wong et al., 2016). Beyond fungi, WjAMP1 from wasabi (*Wasabia japonica*) suppressed bacterial growth, including *E. coli* (IC_50_ ~ 27.5 μg mL^−1^) and *Pseudomonas cichorii* (IC_50_ ~ 13.8 μg mL^−1^) (Kiba et al., 2003). Hevein-like AMPs also contributed to insect resistance, as seen with the MLX56 protein from mulberry, which exhibited antagonistic activity against herbivores like *Spodoptera litura* larvae and *Henosepilachna vigintioctopunctata* (Murata et al., 2021). Additionally, WAMP2-derived peptides enhanced the efficacy of commercial fungicides like Folicur^®^ EC 250, achieving synergistic suppression of fungal spore germination (≥ 90%) (Odintsova et al., 2020).

#### 2.4.4 Roles of hevein-like AMPs in abiotic stress alleviation

In addition to biotic stress, hevein-like AMPs contribute to abiotic stress responses, particularly salinity stress. The expression of *wamp* genes encoding WAMP1 and WAMP2 in *T. kiharae* is significantly induced by elevated NaCl levels (100–200 mM), suggesting a role in mitigating salinity stress (Andreev et al., 2012). Furthermore, transgenic asparagus plants expressing a hevein-like gene show enhanced resistance to stem wilt, accompanied by increased activities of antioxidant enzymes such as CAT, superoxide dismutase (SOD), and phenylalanine ammonia lyase (PAL), and reduced malondialdehyde levels, indicating a broader role in oxidative stress alleviation (Chen et al., 2019).

#### 2.4.5 Applications of hevein-like AMPs genes in transgenic plant development

Hevein-like AMP genes have been extensively utilized in transgenic plant development to enhance resistance to a wide range of biotic stresses, particularly against fungal pathogens and herbivorous insects. As the primary mechanism of hevein-like AMPs is their ability to bind to chitin, a major component of fungal cell walls and insect skeletons, the core function of these peptides does not directly contribute to abiotic stress tolerance. Their application across different crop species demonstrates their broad potential. Several hevein-like genes have been successfully used to combat fungal diseases. The *pnAMP-h2* gene from *Pharbitis nil* conferred improved resistance to *Phytophthora parasitica* in tobacco (Koo et al., 2002), while the *amp1* and *amp2* genes from *T. kiharae* significantly reduced lesion areas caused by *Phytophthora infestans* in tomatoes (Khaliluev et al., 2011). Similarly, the *SmAMP2* gene from *Stellaria media* and a hevein-like gene in asparagus both enhanced resistance to fungal pathogens, including *Phoma asponaqi* and the agent of stem wilt, respectively (Odintsova et al., 2009; Chen et al., 2019). Beyond fungi, hevein-like AMPs also offer protection against insect pests. The *MLX56* gene in transgenic tomatoes provided resistance to herbivorous insects, highlighting its potential as a biological alternative to traditional crop protection methods (Murata et al., 2021). These applications collectively underscore the critical role of hevein-like AMPs in developing resilient, disease-resistant crops by targeting distinct biotic threats.

From the structural stability and direct antimicrobial activity of hevein-like AMPs—which utilize disulfide bonds and chitin-binding domains for pathogen recognition—attention shifts to systemins, a class of peptide hormones that amplify plant defense responses through signaling cascades, notably via the JA pathway. This progression from direct antimicrobial effectors to systemic signaling molecules underscores the functional diversification of AMPs in plant immunity, contributing not only to resistance against biotic stressors but also to enhanced tolerance of abiotic challenges.

### 2.5 Systemins

#### 2.5.1 Family and structure

Systemin (SYS) is an 18-amino-acid peptide hormone derived from a 200-amino-acid precursor, prosystemin (PS), in plants, notably within the Solanaceae family (Zhang et al., 2020). SYS is processed from PS and functions as a long-distance signaling molecule, capable of traveling 40 cm from the injection site to the plant apex at a rate of 2.5 cm h^−1^ in tomato plants (Mucha et al., 2019). Hydroxyproline-rich systemins (HypSYS) are related glycopeptides, first identified in Solanaceae species such as tobacco (*N. tabacum*) and tomato (*Solanum lycopersicum*) (Pearce et al., 1991). HypSYS peptides are small, defense-signaling molecules characterized by hydroxyproline modifications, which enhance their stability and activity. In petunia (*Petunia hybrida*), HypSYS peptides, termed PhHypSYS, exhibited distinct functional roles compared to their tobacco and tomato counterparts (Pearce et al., 2007).

#### 2.5.2 Mechanisms of antimicrobial and defense action of systemin and prosystemin

SYS and HypSYS peptides activate plant defense responses primarily through the JA signaling pathway. In tomato, SYS enhances resistance to necrotrophic fungi like *Plectosphaerella cucumerina* by upregulating JA-biosynthesis gene *LOX2* and JA-responsive genes such as *JASMONATE ZIM-DOMAIN PROTEIN 10* (*JAZ10*), *DEFENSIN1.2* (*PDF1.2*), and *DEFENSIN1.3* (*PDF1.3*) (Zhang et al., 2018; Pastor-Fernández et al., 2022). SYS also interacts with membrane receptors *BAK1* and *BIK1*, which are markers of pattern-triggered immunity (PTI), showing elevated expression only under pathogen challenge, suggesting a unique perception mechanism distinct from classical damage-associated molecular patterns (Lin et al., 2014). Additionally, SYS-treated plants exhibited increased hydrogen peroxide (H_2_O_2_) production following flagellin 22 (flg22) treatment, amplifying defense responses (Zhang et al., 2018). HypSYS peptides from tobacco and tomato, induced defense genes against herbivores, while PhHypSys in petunia upregulated *defensin I*, enhancing pathogen resistance (Pearce and Ryan, 2003; Pearce et al., 2007).

#### 2.5.3 Roles in biotic stress mitigation

SYS and HypSYS peptides are pivotal in mitigating biotic stresses, including fungal, bacterial, and insect challenges. SYS enhanced resistance to necrotrophic fungi such as *B. cinerea* in *Arabidopsis* and tomato by activating JA-responsive defenses, reducing fungal colonization (Coppola et al., 2019). Exogenous SYS application to tomato plants restricts *Spodoptera littoralis* larval growth across generations and attracts natural enemies through volatile compound emission. Similarly, PS application increases *S. littoralis* mortality and suppresses *B. cinerea* colonization (Molisso et al., 2022). HypSYS peptides from tobacco and tomato induce anti-herbivore defenses, such as protease inhibitors, while PhHypSYS in petunia targets pathogen defenses via *defensin I* upregulation, demonstrating functional diversity within the HypSYS family (Pearce and Ryan, 2003; Pearce et al., 2007). SYS treatment increased the expression of pathogenesis-related (PR) gene *PR1* and *PR10*, as well as the PTI market genes *Pti5* and *GRAS2*. It also activated the MAPK signaling pathway, with peak MAPK observed at 3 hours after treatment. In addition, the H_2_O_2_ content in SYS-treated tomato fruits reached the highest level at 12 hours (Wang et al., 2024).

#### 2.5.4 Roles of SYS and PS in abiotic stress alleviation

While the primary role of SYS and HypSys is in biotic stress response, their involvement in abiotic stress alleviation is less direct but notable. The activation of JA-signaling pathways by SYS and HypSys can indirectly enhance resilience to oxidative stress, as JA-responsive genes like *glutathione S-transferase* (*GST*) contribute to detoxification and attenuation of oxidative damage (Gullner et al., 2018). Additionally, the upregulation of heat shock proteins (*HSPs*) in SYS- or PS-treated plants under biotic stress conditions suggests a potential overlap with abiotic stress responses, as HSPs stabilize proteins under various stresses (Corrado et al., 2016; ul Haq et al., 2019). However, specific roles in abiotic stresses like salinity or drought require further investigation.

#### 2.5.5 Applications of SYS and PS genes in transgenic plant development

The genes encoding SYS and PS have been leveraged in transgenic plant development to enhance resistance to a range of biotic stresses, particularly against insects and fungal pathogens. Overexpression of the tomato *PS* gene in *Arabidopsis* conferred higher resistance to the necrotrophic fungus *B. cinerea* by modulating JA-responsive genes (Zhang et al., 2018). Further research on a truncated *PS* (*tPS*) cDNA, which lacks the SYS sequence, showed its remarkable effectiveness in two different host plants. In tomato, it resulted in reduced larval growth and higher mortality of the herbivorous insect *S. littoralis* and suppressed necrosis from *B. cinerea* (Molisso et al., 2022). A similar transformation in tobacco demonstrated that *tPS* enhances resistance to *B. cinerea* by upregulating a suite of defense genes, including heat shock proteins (HSP), glutathione S-transferases (GST), and proteinase inhibitor II (PI II) (Corrado et al., 2016). These findings show that both the full PS protein and its truncated version act as potent signaling molecules that fortify plant defenses through different pathways. Extending the theme of peptide-mediated signaling seen in SYSs, which amplify defense through JA pathways, cyclic dipeptides (CDPs) represent an even more compact and stable class of AMPs. These small molecules provide direct antimicrobial effects while also inducing resistance mechanisms, akin to those seen in hevein-like AMPs and SYSs. This dual functionality reinforces the interconnected roles of diverse AMPs in holistic plant stress management and transgenic innovation. **Table 1** summarizes the application of SYS and PS genes, along with other AMP genes, in transgenic engineering strategies aimed at improving plant tolerance to both biotic and abiotic stresses.

**Table 1:** AMPs-based transgenic plants.

Gene	Origin	Transgenic organisms	Promoter	Targeting pathogens or abiotic agents	Transformation method	References
Thionins						
*α-thionin*	Barley (*H. vulgare* L.)	Tobacco (*N. tabacum* L.)	*CAMV35S*	*P. syringae* pv. *tabaci* 153 *P. syringae* pv. *syringae*	Leaf-disc infection	(Carmona et al., 1993)
β-purothionin	Wheat (*T. aestivum* L.)	*A. thaliana*	*Carbonic anhydrase (CA)* promoter	*P. syringae* strain DC3000 *F. oxysporum* f. sp. *matthiolae*	Vacuum infiltration method	(Oard and Enright, 2006)
Hordothionin	Barley (*H. vulgare*)	Apple (*Malus domestica*)	*CaMV35S*	*P. syringae* pv. *tobacco* *P. syringae* pv. *syringae*	*Agrobacterium tumefaciens* AGL0	(Krens et al., 2011)
α-hordothionin	Barley (*H. vulgare* L.)	Sweet potato (*I. batatas* (L.) Lam.)	*E12Ω*	*Ceratocystis fimbriata*	*A. tumefaciens*	(Muramoto et al., 2012)
Modified thionin (*Mthionin*)	Citrus (*Citrus* L.)	Carrizo citrange (*Citrus triptera* x *C. sinensis*)	Double *35S* (*D35S*)	*Candidatus Liberibacter asiaticus* (Las) *X. citri*	*A. tumefaciens* EHA105	(Hao et al., 2016)
Modified thionin (*Mthionin*)	*A. thaliana*	*A. thaliana*	Double *35S* (*D35S*)	*F. graminearum*	*Agrobacterium*-mediated floral dip method	(Hao et al., 2020)
*Thio-60* and *Thio-63*	*A. thaliana*	*Paulownia tomentosa*	*SP6*	*E. carotovora* *Pseudomonas aeruginosa*	Chitosan nanoparticles	(Hussein, 2020)
*Thio-60*	*A. thaliana*	Onion (*Allium cepa* L.)	*SP6*	*A. niger*	Chitosan nanoparticles	(Tawfik et al., 2022)
*Thio-60*	*A. thaliana*	Date palm (*Phoenix dactylifera* L.)	*SP6*	*F. oxysporum*	Chitosan nanoparticles	(Allah et al., 2023)
Defensins						
*OsDEF7*, *OsDEF8*	Rice (*O. sativa* L.)	*Rosetta-gami E. coli* (DE3)	*Tac*	*Xanthomonas oryzae* pv. *oryzae* *X. oryzae* pv. *oryzicola* *E. carotovora* subsp. *atroseptica*	Not mentioned	(Tantong et al., 2016)
*AtPDF1.1*	*A. thaliana* Col-0	*A. thaliana*	*CAMV35S*	*Pectobacterium carotovorum* subsp. *carotovorum*	*Agrobacterium*-mediated floral dip method	(Hsiao et al., 2017)
*Ca-AFP*	Chickpea (*Cicer arietinum* L.)	*A. thaliana*	*CAMV35S*	Water-deficit stress	*Agrobacterium*-mediated floral dip method	(Kumar et al., 2019)
*ZmDEF1*	Maize (*Z. mays* L.)	Maize (*Z. mays* L.)	*pBetaPhaso*	*Sitophilus zeamais* Motsch	*A. tumefaciens* C58	(Vi et al., 2019)
*PnDEFL1*	*Panax notoginseng*	*A. cepa* L., *N. tabacum* L.	*CaMV35S*	*Fusarium solani* *F. oxysporum* *Botrosphaeria dothidea* *S. sclerotiorum*	*A. tumefaciens* EHA105	(Wang et al., 2019)
*PtDef*	*Populus trichocarpa*	*P. trichocarpa*	*CAMV35S*	*Septotis populiperda*	*A. tumefaciens* EHA105	(Wei et al., 2019)
*MsDef1*	*Medicago sativa*	*N. tabacum*	*M24*	*P. aeruginosa* *R. solanacearum* *X. campestris* *A. niger* *Pyricularia oryzae* *R. solani* *P. syringae* pv *tabaci*	*A. tumefaciens* GV3850	(Deb et al., 2020)
*CAL2*	Rice (*O. sativa* L.)	*O. sativa* L. var. ZH11, *A. thaliana*	*CAMV35S*	Cd detoxification	*Agrobacterium*-mediated floral dip method	(Luo et al., 2020)
*α-TvD1*	Shrub (*Tephrosia villosa* (L.) Pers)	*N. tabacum*	*CAMV35S*	*P. parasitica* var. *nicotianae* *A. alternata* *R. solani* *S. litura*	*A. tumefaciens* LBA4404	(Sharma et al., 2020)
*Chitinase I*, *defensin*	*Solanum tuberosum chitinase I*, *Vigna radiata defensin*	Tea (*Camellia sinensis* L.)	*CAMV35S*	Blister blight (*Exobasidium vexans*)	*A. tumefaciens* LBA4404	(Singh et al., 2020)
*NmDef02*	*Nicotiana megalosiphon*	Soybean (*Glycine max* L.)	*CAMV35S*	*Phakopsora pachyrhizi* *Colletotrichum truncatum*	Bombardement	(Soto et al., 2020)
*pgDEF*	*Panax ginseng*	*A. thaliana*	*CAMV35S*	*F. solani*	*A. tumefaciens* AGL0	(Sun et al., 2021)
*Tfgd2-RsAFP2*	*Impatiens balsamina* L.	Pigeonpea (*Cajanus cajan* (L.) Huth)	*CAMV35S*	*H. armigera*	*A. tumefaciens* EHA105	(Nalluri and Karri, 2023)
*NaD1*	*N. alata*	*N. tabacum* cv. Xanthi tobacco	*CAMV35S*	Drought stress	*A. tumefaciens* GV3101	(Royan et al., 2023)
*RsAFP2*	Radish (*Raphanus sativus* L.)	Chickpea (*C. arietinum*)	*CAMV35S*	*F. oxysporum* f. sp. *Cicero*	*A. tumefaciens* LBA4404	(Sadhu et al., 2023)
Hevein-like antimicrobial peptides						
*Pn-AMP1*, *Pn-AMP2*	*Pharbitis nil* L.	Tobacco (*N. tabacum*)	*CAMV35S*	*P. parasitica*	*A. tumefaciens* EHA101	(Koo et al., 2002
*Pro-SmAmp1*	Chickweed (*Stellaria media*)	*A. thaliana*	*CAMV35S*	*B. cinerea* *B. sorokiniana*	*A. tumefaciens* AGL0	(Shukurov et al., 2010)
*AMP1*, *AMP2*	Chickweed (*S. media*)	Tomato (*S. lycopersicum* L.)	*CAMV35S*	*P. infestans*	*A. tumefaciens* AGL0	(Khaliluev et al., 2011)
*Pro-SmAMP1*, *Pro-SmAMP2*	Chickweed (*S. media*)	Tobacco (*N. tabacum*) cv. Samsun-NN, *A. thaliana* Col-0	*CAMV35S*	*B. sorokiniana* *Thielaviopsis basicola*	*A. tumefaciens* AGL0	(Shukurov et al., 2012)
*Pro-SmAmp2*	Chickweed (*S. media*)	Potato (*S. tuberosum* L.) var. Yubiley Zhukova	*CAMV35S*, *pro-SmAMP2*	*Alternaria* spp. *Fusarium* spp.	*A. tumefaciens* AGL0	(Vetchinkina et al., 2016)
Hevein-like gene	not mentioned	Asparagus (*Asparagus officinalis* L.) var. Jing Kang 701	*CAMV35S*	*Phoma asponaqi* Sacc	*A. tumefaciens* EHA105	(Chen et al., 2019)
*Pro-SmAmp1*	Chickweed (*S. media*)	Potato (*S. tuberosum* L.) var. Zhukovsky ranny and Udacha	*CAMV35S*	*A. alternata* *Alternaria solani*	*A. tumefaciens*	(Beliaev et al., 2021)
Cyclotides						
*Oak1 (kalata B1)*, *asparaginyl endopeptidase*	*O. affinis*	*N. benthamiana*	*CAMV35S*	*In planta kalata B1* production	*A. tumefaciens* LBA4404	(Poon et al., 2018)
Systemins						
*PS* cDNA	not mentioned	Tomato (*S. lycopersicum* L.)	*CAMV35S*	Systemic signal propagation proteinase inhibitor accumulation	*A. tumefaciens* LBA4404	(McGurl et al., 1994)
*PS* cDNA	not mentioned	Tomato (*S. lycopersicum* L.) cv. Red Setter	*CAMV35S*	*Macrosiphum euphorbiae*) *B. cinerea* *A. alternata* *S. littoralis*	*A. tumefaciens* C5851	(Coppola et al., 2015)
*PS* cDNA	not mentioned	Tomato (*S. lycopersicum* L.)	CAMV35S	Cucumber mosaic virus, Necrosis satRNA, Non-necrogenic mutant “NNmut-satRNA”	*A. tumefaciens* LBA4404	(Bubici et al., 2017)
*PS* cDNA	Tomato	*A. thaliana* Col-0	Shoot- or root-specific promoter, *CAMV35S*	*B. cinerea*	*Agrobacterium*-mediated floral dip method	(Zhang et al., 2018)
*Truncated PS* cDNA	Tomato	Tomato (*S. lycopersicum* L.) cv. Red Setter	*CAMV35S*	*S. littoralis* *B. cinerea*	*A. tumefaciens* C5851	(Molisso et al., 2022)

### 2.6 Cyclic dipeptides in plant defense and stress mitigation

#### 2.6.1 Family and structure

CDPs, also referred to as 2,5-diketopiperazines (DKPs), are the smallest cyclic peptides, characterized by a stable 2,5-DKP ring structure (Mishra et al., 2017). These naturally occurring compounds are found in bacteria, yeast, fungi, plants, and animals. Synthesized as secondary metabolites or protein metabolism byproducts, CDPs are produced via CDP synthases and non-ribosomal peptide synthetases (NRPSs), utilizing aminoacyl-tRNAs as substrates, with additional modifications by tailoring enzymes (Fizza et al., 2025). Approximately 100 CDP synthases have been identified, generating 75 of the 210 possible natural CDPs. The cyclization process, involving macrolactomization and macrolactamization, enhances their resistance to peptidase degradation, rendering CDPs highly stable and promising for applications in medicine, industry, and agriculture (Bechtler and Lamers, 2021).

#### 2.6.2 Mechanisms of CDPs in biotic stress mitigation

CDPs enhance plant resistance to biotic stresses through direct pathogen inhibition, insect and nematode deterrence, and induction of systemic resistance. CDPs such as cyclo(L-Phe-L-Pro) and cyclo(L-Phe-trans-4-OH-L-Pro) from *Lactobacillus plantarum* MiLAB 393 inhibited fungi including *Fusarium* sp*orotrichioides*, *Aspergillus fumigatus*, and *Kluyveromyces marxianus* (Ström et al., 2002). Similarly, *Bacillus* sp. strain N produced CDPs like cyclo(L-Pro-L-Leu), cyclo(D-Pro-L-Leu), and cyclo(D-Pro-L-Tyr), which suppressed *Penicillium expansum* (MIC ~ 4 µg mL^−1^), outperforming the fungicide Bavistin, and bacteria such as *Bacillus subtilis* and *Escherichia coli* (Kumar et al., 2012). Cyclo(L-Pro-L-Asp) exhibited potent antifungal activity against *Aspergillus flavus* (MIC ~ 16 µg mL^−1^) and *Trichophyton rubrum* (MIC ~ 2 µg mL^−1^), surpassing standard fungicides (Challa et al., 2014). Cyclo(L-Val-L-Pro) and cyclo(L-Phe-L-Pro) from *Lactobacillus plantarum* LBP-K10 inhibited *Ganoderma boninense*, a major oil palm pathogen (Kwak et al., 2014). Additionally, cyclo(L-Leu-L-Pro) from *Achromobacter xylosoxidans* suppressed aflatoxin production in *Aspergillus* species by targeting glutathione S-transferase (Yan et al., 2004).

CDPs also confer resistance to insects and nematodes (Sun et al., 2021; Sathya et al., 2016). Cyclo(L-Leu-L-Pro) from *Bacillus gaemokensis* PB69 enhanced cucumber resistance to *Pseudomonas syringae* pv. *lachrymans* and increased *Spodoptera litura* larval mortality (Song et al., 2017). Cyclo(L-Pro-L-Ile) from *Bacillus thuringiensis* JCK-1233 suppressed pine wilt disease caused by *Bursaphelenchus xylophilus* in pine seedlings by modulating defense gene expression and preventing hypersensitive reactions (Park et al., 2020). Furthermore, CDPs act as elicitors of induced systemic resistance (ISR). Cyclo(L-Pro-L-Pro) and cyclo(D-Pro-D-Pro) induced resistance in *Nicotiana benthamiana* against *Phytophthora nicotianae* and Tobacco mosaic virus via SA-mediated pathways, triggering stomatal closure, H_2_O_2_ production, and nitric oxide accumulation (Wu et al., 2017). Cyclo(L-Leu-L-Pro) from *Bacillus vallismortis* BS07 activated SA-dependent defenses in chili peppers, reducing disease severity from *Pectobacterium carotovorum* and *Phytophthora capsici* (Noh et al., 2017).

The stability, bioactivity, and simple chemical structures of CDPs make them promising for transgenic plant development. Genes encoding CDP synthases could be integrated into crop genomes to produce antifungal CDPs like cyclo(L-Pro-L-Leu), enhancing resistance to *Fusarium* or *Aspergillus* spp (Boecker et al., 2018). The heat stability and resistance to enzymatic degradation of CDPs ensure their efficacy in field conditions, supporting their use in engineering crops with enhanced biotic stress tolerance (Zhao et al., 2021). Additionally, CDPs’ role in inducing ISR via SA or JA pathways could be leveraged to develop transgenic plants with enhanced systemic immunity (Solis-Ortiz et al., 2022). In summary, CDPs mitigate biotic stresses in crops such as cucumber, chili pepper, pine, and oil palm through direct antimicrobial action, toxin suppression, and SA-mediated ISR, offering versatile mechanisms for enhancing plant immunity against diverse pathogens and pests.

#### 2.6.3 Roles of antimicrobial peptides in abiotic stress alleviation

**Figure 5:**
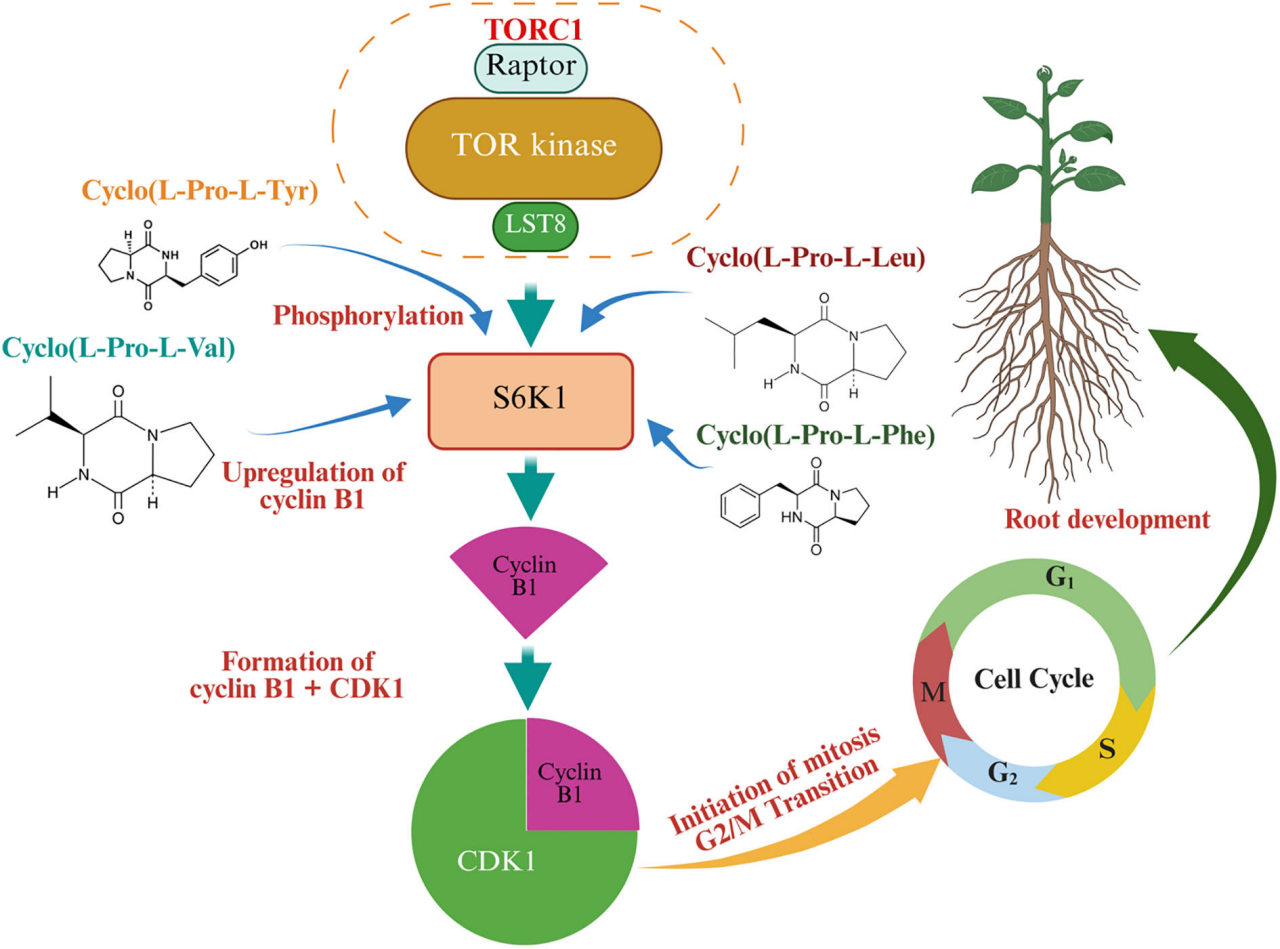
Effects of cyclic dipeptides on root cell proliferation via mitosis regulation.

**Figure 6:**
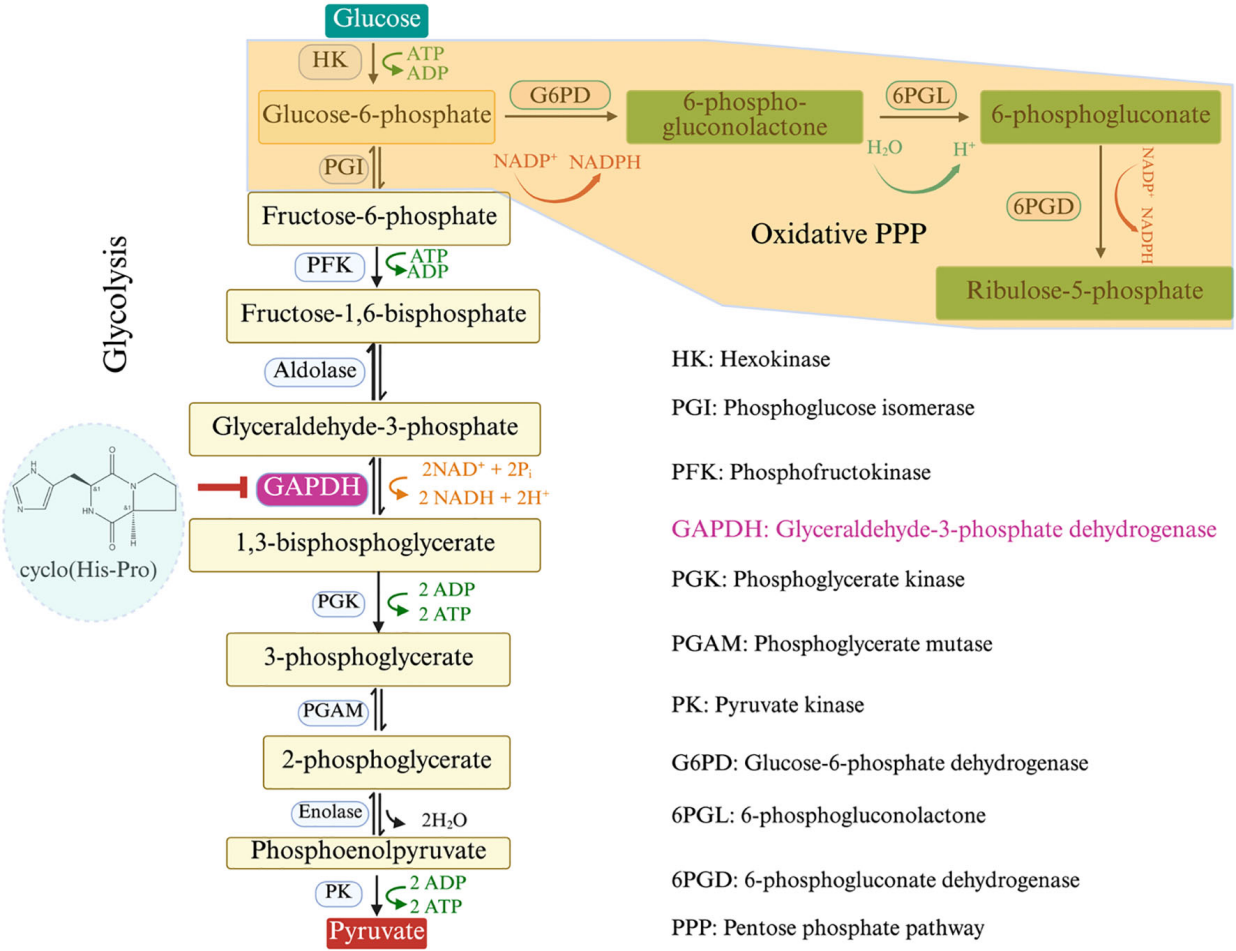
Metabolic pathways of glycolysis and the oxidative pentose phosphate pathway in plants. **Figure 5** illustrates the metabolic pathways of glycolysis and the oxidative pentose phosphate pathway (PPP) in plants, highlighting the interconversion of key intermediates and the enzymes involved. The inhibition of key enzyme glyceraldehyde-3-phosphate dehydrogenase in the glycolysis (on the left) navigates the carbon flux towards the oxidative PPP (on the right), boosting NADPH production for redox balance. **Figure 6** was conceptualized based on the mechanisms described in (Minen et al., 2025).

While research on CDPs’ roles in abiotic stress alleviation is limited, emerging evidence suggests their involvement. A mixture of CDPs (cyclo(L-Pro-L-Tyr), cyclo(L-Pro-L-Val), cyclo(L-Pro-L-Leu), cyclo(L-Pro-L-Phe)) produced by *P. aeruginosa* has been shown to enhance *Arabidopsis* growth and root system architecture (González-López et al., 2021). This is achieved by increasing the phosphorylation of the S6 ribosomal protein kinase (S6K), a downstream substrate of the target of rapamycin (TOR) kinase (**Figure 5**). The TOR pathway is a central hub regulating plant growth, cell proliferation, and metabolism, and its modulation by CDPs suggests a mechanism for improving stress resilience through post-translational modifications (Shi et al., 2018; Caldana et al., 2019). Furthermore, recent studies shed light on the intricate interplay between CDPs and metabolic pathways involved in abiotic stress tolerance. For example, the apparent controversy regarding the role of glyceraldehyde-3-phosphate dehydrogenase (GAPDH) in plant salinity stress tolerance—where some studies report that its upregulation enhanced tolerance (Qiu et al., 2025; Zhang et al., 2024), while others suggest its inhibition by cyclo(His-Pro) improved tolerance (Minen et al., 2025)—can be reconciled by considering the distinct roles, isoforms, and regulatory mechanisms of GAPDH in plant metabolism, as well as the context-specific effects of salinity stress. Upregulation of plastidial GAPDH (pGAPDH) supports photosynthesis and energy production, critical for maintaining growth and osmolyte synthesis under salinity (Li et al., 2019). Conversely, inhibition of cytosolic GAPDH (cGAPDH) by cyclo(His-Pro) redirects carbon flux from glycolysis to the pentose phosphate pathway (PPP), enhancing nicotinamide adenine dinucleotide phosphate (NADPH) production and antioxidant defense, which is vital for mitigating oxidative stress (Minen et al., 2025) (**Figure 6**). These mechanisms are not mutually exclusive but reflect a dynamic metabolic balance tailored to specific stress conditions, tissues, and plant species. For instance, under normal conditions, the microbial inoculant *Priestia megaterium* BP01R2, which likely produces CDPs, induced lateral root formation, created more root hairs, increased fresh weight, and promoted main root length (Hung et al., 2024). Under salinity stress, *P. megaterium* BP01R2 mitigated adverse effects by reducing chlorotic and curled leaves and minimizing the decline in root development. The expression pattern of *P. megaterium* BP01R2 -inoculated *Arabidopsis* plants under salt stress resembled that of control plants rather than NaCl-treated plants, suggesting a significant stress-mitigating effect. Under stress-free conditions, genes related to root cell differentiation, root morphology, cytoskeleton, and various enzyme activities (ATPase, helicase, ligase) were upregulated, aligning with enhanced plant growth. Under stress conditions, genes involved in cell death, hypersensitive response, JA biosynthesis and response, ET response, MAPK signaling, and glutathione metabolism were also upregulated, indicating a broader activation of stress response pathways (Hung et al., 2024). Intriguingly, the exogenous application of cyclo(L-Ala-Gly), which was identified in the metabolomic data of *P. megaterium* BP01R2, mirrored the performance of its host bacterium BP01R2 in alleviating salinity stress in *Arabidopsis* (Hung et al., 2024), suggesting potential applications of these small molecules as biostimulants in agriculture. These initial studies paved the way for a partial understanding of the underlying mechanisms behind CDPs’ properties in abiotic stress mitigation. However, further investigations integrating transcriptomic, proteomic, and metabolomic approaches are needed to fully elucidate the complex mechanisms underlying CDPs’ contributions to abiotic stress tolerance, such as drought, salinity, or temperature extremes.

**Figure 5** illustrates the molecular mechanisms involved in root development, with a focus on the Target of Rapamycin (TOR) signaling pathway. TOR kinase complex, consisting of TOR kinase, Raptor, and LST8, functions as the central regulator. The complex is induced by cyclic dipeptides [e.g., Cyclo(L-Pro-L-Tyr), Cyclo(L-Pro-L-Val), Cyclo(L-Pro-L-Leu), and Cyclo(L-Pro-L-Phe)], and subsequently facilitates the phosphorylation process to activate S6K1. S6K1 upregulates the expression of Cyclin B1, which leads to the formation of cyclin B1 and cyclin-dependent kinase 1 (CDK1). The Cyclin B1–CDK1 complex drives the transition from the G2 phase to the M phase (mitosis) in the cell cycle, thereby promoting root development in plants. **Figure 5** was conceptualized based on the mechanisms described in (Caldana et al., 2019; González-López et al., 2021).

## 3 Future perspectives

From an agricultural perspective, AMPs have been extensively studied for their antifungal and antibacterial properties, with significant potential for enhancing crop resistance to biotic stresses. Recent studies also highlight salient roles of AMPs in abiotic stress responses. However, the mechanisms underlying AMPs’ involvement in abiotic stress responses remain underexplored, which necessitate comprehensive evidence on molecular features, signaling pathways, phytohormone interactions, and field-based assessments to validate their efficacy in crops. The potential of AMPs as biocontrol agents, either through direct application or transgenic approaches, is promising, given their stability and broad-spectrum activity. Nevertheless, utilizing AMPs as elicitors to induce abiotic stress tolerance requires further investigation to elucidate their modes of action and optimize their application. Bridging this knowledge gap is critical for developing resilient crop varieties to address rising environmental challenges. Additionally, the release of bioactive AMPs from genetically modified crops raises concerns for human health and ecosystems. The high stability of AMPs suggests persistence in soil and water, therefore, thorough assessments of their environmental fate, interactions with soil microbiota, and degradation dynamics, are highly recommended, to ensure safe deployment.

To advance the application of AMPs in agriculture and address current knowledge gaps, the following research directions and recommendations are proposed:

- Elucidate Molecular Mechanisms: Investigate the signaling pathways and molecular interactions of AMPs in abiotic stress responses, focusing on their crosstalk with phytohormones like JA, ABA, SA to uncover synergistic effects.
- Expand Field-Based Studies: Conduct greenhouse and in-field trials to evaluate AMP efficacy across diverse crop species under combined biotic (pathogens, insects) and abiotic (salinity, drought, heavy metals) stresses, ensuring translational relevance.
- Assess Environmental Impact: Perform comprehensive studies on the persistence, degradation, and ecological interactions of AMPs in soil and water, including their effects on non-target organisms and microbial communities, to ensure biosafety.
- Integrate Multi-Stress Resistance: Explore gene stacking strategies combining AMPs (e.g., SYS with *SOS1*, NaD1 with *HSP90*) to engineer crops with simultaneous resistance to biotic and abiotic stresses, balancing efficacy with yield stability.

These directions aim to harness AMPs potential for sustainable agriculture while addressing ecological and safety concerns, paving the way for resilient crop systems amidst global environmental challenges.
